# Metabolomic Hallmarks of Obesity and Metabolic Dysfunction-Associated Steatotic Liver Disease

**DOI:** 10.3390/ijms252312809

**Published:** 2024-11-28

**Authors:** Diren Beyoğlu, Yury V. Popov, Jeffrey R. Idle

**Affiliations:** 1 Department of Pharmaceutical and Administrative Sciences, College of Pharmacy and Health Sciences, Western New England University, Springfield, MA 01119, USA; diren.beyoglu@wne.edu; 2 Division of Gastroenterology, Hepatology and Nutrition, Beth Israel Deaconess Medical Center, Harvard Medical School, Boston, MA 02215, USA; ypopov@bidmc.harvard.edu

**Keywords:** obesity, MASLD, amino acids, energy metabolites, gut microbiota metabolites, acylcarnitines, LPC/PC, LPE/PE, fatty acids, bile acids

## Abstract

From a detailed review of 90 experimental and clinical metabolomic investigations of obesity and metabolic dysfunction-associated steatotic liver disease (MASLD), we have developed metabolomic hallmarks for both obesity and MASLD. Obesity studies were conducted in mice, rats, and humans, with consensus biomarker groups in plasma/serum being essential and nonessential amino acids, energy metabolites, gut microbiota metabolites, acylcarnitines and lysophosphatidylcholines (LPC), which formed the basis of the six metabolomic hallmarks of obesity. Additionally, mice and rats shared elevated cholesterol, humans and rats shared elevated fatty acids, and humans and mice shared elevated VLDL/LDL, bile acids and phosphatidylcholines (PC). MASLD metabolomic studies had been performed in mice, rats, hamsters, cows, geese, blunt snout breams, zebrafish, and humans, with the biomarker groups in agreement between experimental and clinical investigations being energy metabolites, essential and nonessential amino acids, fatty acids, and bile acids, which lay the foundation of the five metabolomic hallmarks of MASLD. Furthermore, the experimental group had higher LPC/PC and cholesteryl esters, and the clinical group had elevated acylcarnitines, lysophosphatidylethanolamines/phosphatidylethanolamines (LPE/PE), triglycerides/diglycerides, and gut microbiota metabolites. These metabolomic hallmarks aid in the understanding of the metabolic role played by obesity in MASLD development, inform mechanistic studies into underlying disease pathogenesis, and are critical for new metabolite-inspired therapies.

## 1. Introduction

Using a modified Delphi technique [1], a panel of 236 experts from 56 countries recently introduced the nomenclature, metabolic dysfunction-associated steatotic liver disease (MASLD), and metabolic dysfunction-associated steatohepatitis (MASH) [2], building on the earlier consensus version of metabolic dysfunction-associated fatty liver disease (MAFLD) [3,4]. Steatotic liver disease was selected as an all-embracing term for the various etiologies of steatosis. The term steatohepatitis was thought to be an important pathophysiological construct that should be preserved [2]. This new nomenclature is intended to replace nonalcoholic fatty liver disease (NAFLD), alcohol-related liver disease (ALD) and nonalcoholic steatohepatitis (NASH).

MASLD is characterized by fat accumulation accounting for a minimum of 5% of hepatic mass, a condition referred to as steatosis. This deduction is ascertained through biopsy/histology or imaging of the liver. MASLD can progress to the more aggressive MASH, which is characterized by the presence of hepatic steatosis surpassing 5%, accompanied by indications of hepatocellular injury, such as hepatocyte ballooning, inflammation, and advanced fibrosis. MASH may subsequently progress to cirrhosis that increases the risk of hepatocellular carcinoma (HCC) by about 2% per annum [5]. Recently, the worldwide prevalence of MASLD was estimated to be 32.4% [6], making it the most widespread global liver disease [7]. MASLD is also more prevalent in individuals with cardiovascular/metabolic risk factors, including type 2 diabetes (T2DM), obesity, hypertension, dyslipidemia, and metabolic syndrome [8,9]. Moreover, metabolic syndrome is thought more likely to arise in persons with MASLD than those without it [10]. In one study, 74.6% of patients with metabolic syndrome had MASLD [11]. The estimated global prevalence of MASLD in patients with T2DM was 55.5% and in patients with obese T2DM was 85.0% [9]. Meta-analyses provided statistically significant, adjusted hazard ratios for risk of incident severe liver disease outcomes of 2.25 and 1.20 for T2DM and obesity (BMI > 30 kgm^−2^), respectively [8]. Evidence indicates that obesity significantly increases the risk of developing MASLD with an odds risk (OR) of 4.6, cirrhosis with an OR of 4.1, and HCC with an OR of 1.89 [12]. With the global epidemic in obesity and T2DM, it is estimated that the prevalence of MASLD will continue to rise at an alarming rate [6], yet further exacerbating this major public health crisis. In the United States, MASLD was calculated to have resulted in 4.5 million persons with advanced liver fibrosis and 600,000 with cirrhosis [13]. The economic burden of MASLD is considerable, exceeding $100 billion in the USA alone, with liver transplantation being a substantial contributor to healthcare costs [7].

The detailed mechanism by which MASLD progresses to MASH and HCC is not completely understood, although considerable progress has been made of late. The pathophysiology of MASLD progression has been investigated with various propositions appearing in the literature. One involved the role of liver sinusoidal epithelial cells (LSECs), the most abundant non-parenchymal cells in the liver that serve essential functions in the transfer of nutrients, lipids, and lipoproteins. The loss of the normal fenestrations of LSECs during capillarization has been reported to proceed fibrosis in MASLD [14]. The transport of exactly which molecules across LSECs is affected by capillarization is unclear. Second, thrombospondin 2 (TSP2) is a secreted glycoprotein that is involved in cell-to-cell and cell-to-extracellular matrix (ECM) interactions by binding with cell surface receptors, growth factors, cytokines, or components of ECM proteins [15]. Serum TSP2 was reported to be associated with both the severity and progression of the metabolic syndrome and MASLD [11]. Third, there appears to be compelling evidence that the severity of MASLD is related to endocrine dysfunction. It is believed that low levels of sex hormones, growth hormone and thyroid hormone promote the development and progression of MASLD [16]. Fourth, dysregulation of the urea cycle leading to excess cellular ammonia concentrations has been shown to occur in MASLD, due to increased methylation of urea cycle genes. Moreover, progression from MASLD to MASH was said to involve this hyperammonemia state [17]. An alternative mechanism for excess cellular ammonia generation has been proposed, that of upregulation of glutaminase 1, the enzyme that converts glutamine into glutamate plus ammonia. The hepatic glutaminase 1 overexpression was related to the lipopolysaccharide (LPS)/Toll-like receptor 4 (TLR4) axis [18]. LPS, also known as endotoxin, is a component of the outer membrane of Gram-negative bacteria where it occupies up to 80% of the bacterial surface in *E. coli* and *Salmonella* and from where it can be secreted in outer membrane vesicles [19]. Passage across the gut permits LPS to interact with TLR4, predominantly on monocytes, macrophages, and dendritic cells, leading to activation of innate immunity with the release of proinflammatory cytokines and chemokines [20]. TLR4 is also expressed in all parenchymal and nonparenchymal cells of the liver [21], whereby its activation by LPS leads to the upregulation of glutaminase 1 and subsequent elevated ammonia levels and progression of MASLD to MASH [18].

As their names suggest, MASLD and MASH are clearly metabolic disorders. Metabolomics may therefore be a beneficial tool to disclose the metabolic details of both the development of MASLD and its progression to MASH and HCC. Clues were already apparent from animal studies that sought to uncover discrete metabolic changes but without using metabolomic methodologies. For example, an intermittent fasting 5:2 regimen in mice (two nonconsecutive 24 h periods in one week, for 32 weeks) prevented MASH development on a Western diet. The fasting response involved PPARα and glucocorticoid signaling, lowering hepatic triglycerides and steatosis [22]. Metabolomics applied to obesity and MASLD development, together with MASLD progression, is discussed below.

## 2. Metabolomics

### 2.1. Introduction

We have recently given a detailed account of metabolomic methodologies applied to liver disease [23] and have cited examples of how such studies are conducted using both gas chromatography-mass spectrometry (GC-MS) and ultraperformance liquid chromatography-quadrupole time-of-flight mass spectrometry (UPLC-QTOFMS) [24,25,26,27,28,29,30,31,32,33,34,35,36,37,38,39]. Using metabolomics, it is commonplace to screen biofluids, such as urine or serum, for their metabolite composition in disease cases and matched controls in order to find biomarkers for risk prediction [40]. Furthermore, alterations in biological pathways can be detected that provide insights into disease mechanisms [41]. Experimental metabolomic findings may be subject to a “false discovery rate” (FDR) due to multiple testing, as described in detail by Benjamini [42,43]. It was proposed that multiple testing could be corrected for by using a Bonferroni procedure [42,43]. There has been some criticism that multiple testing procedures can reduce statistical power [44,45]. Nevertheless, correction for multiple testing is widely used in metabolomics investigations [46,47,48,49] and is combined with multiple linear regressions methods [50]. Furthermore, many metabolomics studies have issues with metabolite identification, quantitation, and biological interpretation. In particular, many investigators have relied solely on accurate mass and database mining to assign metabolite identity. To address this and other issues, NIH sponsored the Metabolomics Standards Workshop in 2006 [51], which led to the Metabolomics Standards Initiative (MSI) 2007 [52,53], which was revised a decade later [54]. It is now accepted that the gold standard for metabolite annotation, by both mass spectrometry and NMR methodologies, is comparison with an authentic standard. However, using mass spectrometry, putative identifications are often assigned by matching the mass of a feature with compounds in chemical libraries such as Metlin, KEGG, or ChemSpider. This can result in multiple indistinguishable and often incorrect annotations. Comparing the MS/MS spectrum of the feature with MS/MS spectra in databases can yield a higher confidence of annotation. The reader is directed to [55] for a more detailed discussion of metabolite annotation.

Several investigators have combined metabolomics observations with transcriptomic and proteomic data. We were the first to combine metabolomics and transcriptomics in the study of hepatocellular carcinoma, which revealed that, contrary to expectation, the Wnt/β-catenin pathway activated by *CTNNB1* mutation in certain transcriptomic subgroups did not exhibit specific metabolic remodeling to glycolysis over mitochondrial oxidative phosphorylation [26]. In general, metabolomics represents the downstream product of both transcriptomics and proteomics and therefore provides a snapshot of the functional state of the cell at any given time. Such multi-omics approaches, involving genomics, epigenomics, transcriptomics, proteomics, and metabolomics have been discussed in the context of precision obesity management. This novel approach was deemed necessary due to the failure to curtail the accumulating burden of obesity worldwide [56]. Additionally, a recent investigation of the effects of intermittent fasting on fatty liver disease in mice utilized combined proteome, transcriptome, and metabolome analyses to identify that peroxisome-proliferator-activated receptor alpha (PPARα) and glucocorticoid-signaling-induced PCK1 act co-operatively as hepatic executors of the fasting response [22].

One of the ways in which metabolomic data are of practical clinical value is the development of biomarkers that can distinguish disease subtypes, for example in obesity [57]. In MASLD, a very recent Chinese study identified three distinct molecular subtypes by using integrative multi-omics including whole-genome sequencing, proteomics, phosphoproteomics, lipidomics, and metabolomics across a broad range of liver, blood, and urine specimens. These distinct subtypes were thought to have quite different clinical outcomes [58], leading to differential potential therapeutic approaches.

In metabolomic investigations, it can occur that two groups studying the same clinical problem arrive at different results, sometimes generating widely different biomarkers. How does this happen? First, the study populations are likely to be different. Metabolic reactions are recognized as displaying interindividual differences and different populations may express contrasting metabolic capacities. Second, and importantly, almost all investigators use different workflows with dissimilar instrumentation, such as NMR, LC-MS, and GC-MS, as we have recently discussed [23]. The choice of instrumentation can have a profound effect on the results. How the data are analyzed may also influence the findings. It is possible to use multivariate data analysis with methods such as unsupervised PCA or supervised PLS-DA and OPLS-DA [59]. Machine learning procedures such as random forests can be used to analyze metabolomic data [24,60].

For reviews of the clinical application of metabolomics as a noninvasive methodology, the reader is directed to the following references [61,62,63].

### 2.2. Metabolomics of Obesity

The earliest overtures about the value of metabolomics in studying obesity were made by Griffin [64,65]. Experimental studies are reported in Table 1.

#### 2.2.1. Metabolomics of Obesity in Experimental Animals

Table 1 contains six metabolomic studies on obesity conducted in rats. All involved the comparison of obese and lean rats, with metabolomic analyses on blood, plasma, or serum, together with urine, liver, muscle, and adipose tissue [66,67,70,75,87,91]. In general, metabolites elevated in obesity in the rats included various lipids, for example, cholesterol, lysophosphatidylcholines (LPCs), fatty acids (FAs), energy metabolites (glucose, glycerol, lactate, pyruvate, acetate, 2-hydroxyisobutyrate), amino acids (methionine, glycine, cystine, alanine, glutamate), and gut microbiota metabolites (indole-3-carboxylic acid, phenacetylglycine, indoxyl sulfate and glucuronide, *p*-cresol sulfate). Table 1 also contains six metabolomic studies on obesity conducted in mice. Again, all studies involved comparison of obese and lean mice, with metabolomic analyses of liver tissue, serum and urine [68,69,71,77,85,90]. Elevated metabolites in obese mice comprised diverse lipids, including phosphocholines (PCs), LPCs, acylcarnitines, cholesterol, 7-ketodeoxycholic acid, VLDL/LDL, energy metabolites (glucose, glycerol, pantothenic acid, acetoacetate, acetone, citrate, fumarate, 2-oxoglutarate, succinate, 3-hydroxybutyrate, acetate, lactate, pyruvate), amino acids (arginine, tyrosine, glutamine, citrulline, phenylalanine, glycine, alanine, pipecolic acid), other metabolic intermediates (nicotinamide derivatives, uric acid, allantoin, TMAO, serotonin), and gut microbiota metabolites (benzoic acid, phenacetylglycine, phenylpyruvic acid, phenylacetamide).

#### 2.2.2. Metabolomics of Obesity in Humans

The human studies listed in Table 1 are more heterogeneous, eighteen conducted in obese adults, two in obese women with polycystic ovary syndrome (PCOS), one in pregnant women, two in obese adolescents, and five in obese children.

##### Metabolomics of Obesity in Adults

Elevated metabolites in healthy obese adults included serum PC(42:0), glycine, and glutamine [72]; in men and women with abdominal obesity it included serum TG(54:1–3), which correlated with abdominal visceral obesity in women, while serum TG(50:1–5), TG(55:1), and PC(32:0) correlated with abdominal visceral obesity in men [76]. A more complete picture emerged from investigations of nondiabetic obese vs. nondiabetic lean adults [79] and Chinese obese vs. lean young students (age 18–23 years) [80]. The principal elevated serum metabolites in these two reports comprised free fatty acids (14:0, 16:0, 16:1, 18:0, 18:1, 18:2, 18:3, 20:2, 20:5, 22:4, 22:5) and amino acids (lysine, glutamine, proline, threonine, leucine, isoleucine, valine, histidine, alanine, asparagine, phenylalanine), with assorted metabolic intermediates (3-hydroxybutyrate, choline, 3,7-dimethylurate, pantothenate, *myo*-inositol, sorbitol, glycerol, glucose). Another study investigated skeletal muscle and plasma metabolites in obese vs. lean male adults. 2-Oxoglutarate was elevated in obese skeletal muscle, with C3, C4, and C10:1 acylcarnitines elevated in obese plasma [81]. Yet more heterogeneity appeared in a Chinese report of serum from obese vs. normal weight men [82], in which diverse lipids were elevated (acylcarnitine C8:1, FA(20:2), 12-HPETE, 4-hydroxystrone sulfate, LPE(18:1), TxB2) as was the α-amino acid and collagen crosslink hydroxylysylpyridinoline. Later, it became popular to study the serum metabolomics of the response to a caloric challenge in healthy obese (HO), unhealthy obese (UHO), and lean healthy (LH) [84] subjects. The authors concluded, “Minor differences were found in postprandial responses for amino acids between MHO and MUO individuals, while three polyunsaturated FAs (18:1, 18:2, 20:4) showed smaller changes in serum after the meal in MHO individuals compared to MUO. MHO individuals show preserved insulin sensitivity and a greater ability to adapt to a caloric challenge compared to MUO individuals.”. Another such study compared MHO and MUHO persons [95]. MHO patients had elevations in plasma lipids (PC(32:1), PC(38:3)), and amino acids (BCAAs, tyrosine, glutamate). In contrast, MUHO patients displayed different elevated lipids (PC(32:2), PC(34:2), LPC(16:1), acylcarnitine C3), and amino acids (proline). Another variation was the study of severely obese vs. nonobese subjects, conducted in relation to bariatric surgery [86]. Severely obese patients (BMI not declared) had elevated serum VLDL1, elevated amino acids (alanine, BCAAs, tyrosine, phenylalanine), and energy metabolites (pyruvate, citrate, acetoacetate, glucose). Patients were reevaluated 12 months after bariatric surgery and the authors concluded, “Our data indicate that bariatric surgery, irrespective of the specific kind of procedure used, reverses most of the metabolic alterations associated with obesity and suggest profound changes in gut microbiome–host interactions after the surgery.”. An investigation of adult morbid obesity vs. nonobese patients reported that serum had elevated glutamate and 12 ceramides [92].

Metabolic syndrome is a concept used to distinguish “well” from “unwell” obese subjects, and it is a major risk factor for type 2 diabetes and cardiovascular disease [97]. Plasma metabolomics was studied in obese well, obese unwell, and lean well patients. The concept of metabolite changes along a spectrum of metabolic wellness was developed, with worsening health associated with BCAAs, cystine, α-aminoadipate, phenylalanine, leucine, lysine, and acylcarnitine C3. Tyrosine, alanine, and acylcarnitine C3 increased with obesity and metabolic unwellness [97]. Several studies compared obese with nonobese subjects [98,99,100,101,102]. Elevated serum/plasma metabolites included amino acids (phenylalanine, tryptophan, BCAAs, alanine, glutamate, proline, tyrosine, arginine, lysine), various lipids (FA(18:2), LPC(14:0), LPC(16:0), LPC(16:1), PC(32:1), PC(32:2), PC(38:3), HPODE, HODE/EpOME), gut microbiota metabolites (phenylacetamide, phenylpyruvic acid), and various metabolic intermediates (uric acid, carnitine). One group sought to distinguish between the causes of obesity and the effects of obesity [104]. Elevated plasma metabolites said to be related to the cause of obesity were 2-hydroxybutyrate, PC(34:4), PCE(18:1), and acylcarnitine C6. Metabolites related to the effect of obesity were valine, LPC(22:6), and acylcarnitine C18. Elevated glycine and tyrosine were related to both the cause and effect of obesity.

Two studies on obese women with PCOS [74,105] revealed elevated plasma glycerol, FA(18:1), FA(18:2), FA(20:3), FA(20:4), FA(20:5), FA(22:4), FA(22:6), taurocholate, DHEA sulfate, 9,12,13-triHOME, pregnenolone sulfate, and bilirubin. One investigation into obesity in pregnancy utilized obese and nonobese gravidae and reported changes in metabolite levels in the placenta [94], including elevated amino acids (tyrosine, phenylalanine, isoleucine, leucine, serine), metabolic intermediates (uracil, hypoxanthine, glucose 6-phosphate, 3-phosphoglycerate, glycerol, nicotinamide), and FA(16:0).

##### Metabolomics of Obesity in Adolescents

Two studies conducted in obese adolescents vs. normal weight adolescents [73,88] appear in Table 1. No elevated metabolites in the plasma of obese adolescents were reported [73] and in urine, elevated carnitine and various acylcarnitines (C3, OH-C3, C5, C8, C10, C12, C14), aspartate, asymmetric dimethylarginine, and putrescine [88] were found.

##### Metabolomics of Obesity in Children

Childhood obesity is associated with increased risk of glucose intolerance, hypertension, dyslipidemia, insulin resistance, chronic inflammation, hyperuricemia, and nonalcoholic fatty liver disease [106]. Physiological responses to metabolic disturbances would appear to be different in early life [78]. BCAA-related metabolic patterns and androgen (dehydroepiandrosterone sulfate) metabolite-related patterns were associated with childhood obesity, with children of obese mothers having higher BCAA plasma levels [78]. Many metabolites were reported to be elevated in the serum of obese children [89], including several lipids (taurodeoxycholate, glycodeoxycholate, LPC(14:0), LPE(16:0), LPE(18:0), LPE(18:1), LPE(18:2), LPE(20:3), LPS(19:0), LPS(20:4), methylbutyrylcarnitine), amino acids (BCAAs, alanine, proline, tryptophan, phenylalanine, tyrosine, arginine, aspartate), and various metabolic intermediates (threitol, piperidine, pyruvate, lactate, 2-ketoisocaproate). In another child obesity study, only lactate was found to be elevated in plasma [96].

#### 2.2.3. Metabolomic Patterns of Obesity

As shown in Section 2.2.1, circulating metabolites elevated in obese rats and obese mice could be listed under four main headings, (1) diverse lipids, (2) energy metabolites, (3) amino acids, and (4) gut microbiota metabolites. A similar pattern is recapitulated in obese adult humans (Section Metabolomics of Obesity in Adults), with miscellaneous lipids and amino acids dominating the obese adult human serum metabolic phenotype. Concerning (1), the elevated diverse lipids reported in obese adult human serum were dominated by (1a) phospholipids (phosphatidylcholines and lysophosphatidylcholines), free fatty acids, and acylcarnitines. Regarding (2), the elevated energy metabolites, the following have been reported: (2a) ketone bodies (acetoacetate and 3-hydroxybutyrate), (2b) an early indicator of glucose intolerance in nondiabetic patients (2-hydroxybutyrate) [107], and (2c) metabolites involved in energy generation (pantothenate, carnitine, sorbitol, glycerol, glucose, glucose 6-phosphate, 3-phosphoglycerate, pyruvate, lactate, citrate, nicotinamide). In the case of (3), amino acids, obese adult human serum/plasma contained (3a) the nonessential amino acids (glycine, alanine, serine, glutamate, glutamine, proline, asparagine, arginine, cysteine, tyrosine) and (3b) the essential amino acids (lysine, leucine, isoleucine, valine, threonine, histidine, tryptophan, phenylalanine). Among (4), the gut microbiota metabolites in the circulation of adult human obese subjects, phenylacetamide (a metabolomic marker of immune aging [108]) and phenylpyruvate [109] were reported.

The two reports of adolescent obesity (Section Metabolomics of Obesity in Adolescents) were uninformative regarding plasma biomarkers of obesity, but urine contained elevated lipids in the form of multiple acylcarnitines, from C3 to C14. Additionally, increased urinary excretion of aspartate and asymmetric dimethylarginine (ADMA), together with putrescine, was reported. ADMA inhibits nitric oxide (NO) synthesis from arginine and therefore compromises endothelial function, which may lead to coronary artery disease [110]. Putrescine, on the other hand, together with overexpression of ornithine decarboxylase 1 (ornithine → putrescine), has been found to be elevated in relation to the progression of MASLD to MASH [111].

In the case of childhood obesity (Section Metabolomics of Obesity in Children), a wide variety of lipids were elevated in the serum of obese children. These comprised lysoglycerophospholipids (LPC, LPE, LPS) and conjugated bile acids. Moreover, both essential and nonessential amino acids were elevated, together with various metabolic intermediates and energy metabolites. The serum lysoglycerophospholipids found were mainly LPEs, whose pathophysiological role is uncertain. Most reports have come from cultured cells [112].

These obesity metabolomic biomarkers appear to give early indications of impending diabetes, heart disease and even MASLD. The relationship between obesity and MASLD will be explored in more detail in the next section using metabolomic data.

**Figure 1:**
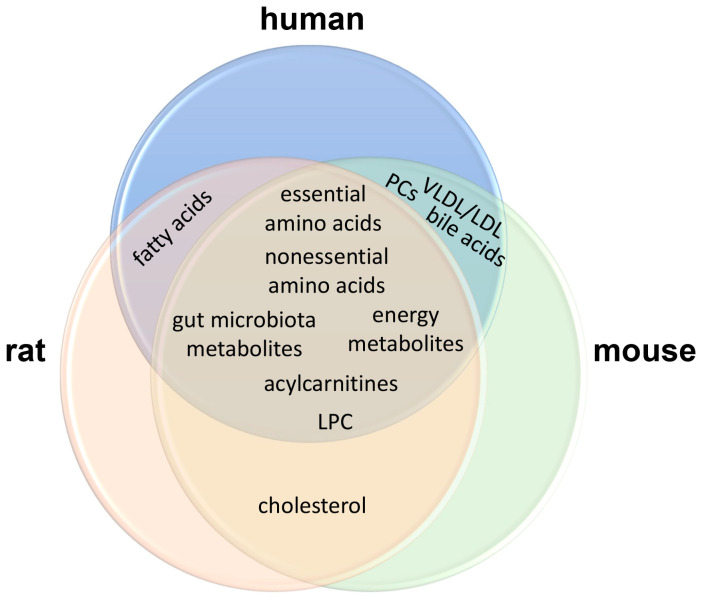
Venn diagram showing the shared elevated obesity metabolomic biomarker groups in human, rat, and mouse.

Figure 1 shows that obese humans, rats, and mice share elevated essential and nonessential amino acids, gut microbiota metabolites, energy metabolites, acylcarnitines, and LPC. None of these three species harbored a uniquely elevated biomarker for obesity. Cholesterol was elevated in rat and mouse serum but not in human serum. Fatty acids were elevated in rat and human serum but not in murine serum. In addition, VLDL/LDL, PCs, and bile acids were enhanced in the sera of humans and mice but not in rats. Overall, the similarities between experimental obesity in rodents and human obesity were greater than their differences in terms of metabolic phenotype.

#### 2.2.4. The Metabolomic Hallmarks of Obesity Preeminent in the Circulation

1. Lysophosphatidylcholines (LPC)
2. Acylcarnitines
3. Essential amino acids
4. Nonessential amino acids
5. Energy metabolites
6. Gut microbiota metabolites

### 2.3. The Metabolomics of MASLD

It should be borne in mind that MASLD was previously called NAFLD and also encompassed ALD (see above). For the purposes of clarity, MASLD, NAFLD, and ALD will all be referred to here as MASLD. As MASLD is a metabolic disorder, metabolomics should be helpful in defining the metabolic granularity of the disease, including the transition from obesity to MASLD. The experimental investigations are reported in Table 2.

Table 2 reviews 51 studies of MASLD metabolomics and its predecessors NAFLD and ALD in various species including humans (26), mice (12), rats (8), hamsters (1), cows (1), geese (1), blunt nose breams (1) and zebrafish (1). Only elevated metabolites that might be used as biomarkers are shown. In only one example (zebrafish), the whole body was examined for metabolites, otherwise plasma/serum (36), urine (6), liver (10), and feces (2) were analyzed. The vast majority of investigations employed either UPLC-QTOFMS or GC-TOFMS and therefore a wide range of metabolites are described.

#### 2.3.1. Metabolomics of MASLD in Humans

Elevated free fatty acids (FFA) in human plasma/serum was an uncommon finding, with only three analyses reporting FFA, which were FA(11:0) and FA(18:3) in adults [113] and FA(18:0), FA (20:2) [139], and FA(20:4) [158] in children and FA(8:0) in adolescents [118]. However, the transport form for FFA, acylcarnitines, was found much more commonly, including acylcarnitine C0, C4 [113], C3 [120], C0, C3, C3DC, C4, C5, C5OH, C8:1, C10, C14OH, C14:1OH, C16:1, C16:2, C18, C18OH, C18:1, C18:2, C20, C20:4 [130], C2, C3 [137], C5, C8, C11OH, C12OH, C12OHDC, C14:1-3OH [142], C4OH, C8OH [148] in adults, C0, C10:2, and C14:1 [125] in adolescents and C6, C8, C10, and C10:2 in children [151]. These findings represent a generalized deficiency in short-chain, medium-chain, and long-chain β-oxidation, that is, the clearance of FFA from the liver by mitochondria. Metabolomic studies have provided evidence from human plasma/serum that the uncleared fatty acids led to the synthesis of triacylglycerides, with prominent levels of TG(52:1), TG(53:1), TG(53:0), TG(58:2), TG(54:5) [126], TG(54:0), TG(54:1), TG(53:0), TG(52:0), TG(50:0), TG(49:0), TG(48:0), TG(46:0), TG(45:1), and TG(44:1) [141], including increased levels of the intermediate monoacylglycerols and diacylglycerols MG(18:1), DG(18:1/18:2), and DG(20:3/20:4) [142]. These last data point to a partially incomplete process of lipogenesis, which occurs predominantly in liver and adipose. Another common group of fatty acid derivatives that did not appear to be elevated in plasma/serum of human MASLD were the cholesterol esters, with only one report citing elevated free cholesterol [160], suggesting an inactivity of the synthetic enzyme acyl-CoA: cholesterol acyltransferase (ACAT) or over activity of the hydrolytic enzyme lysosomal acid lipase (LAL). A specific type of authophagy known as lipophagy, the autophagic breakdown of intracellular lipid droplets [161], does not appear to be properly regulated in fatty liver diseases [162]. The core of lipid droplets that accumulate in the liver during MASLD largely comprises neutral acylglycerides and cholesterol esters [161], while the lipid droplet membrane is principally composed of phosphatidylcholine, followed by phosphatidylethanolamines, phosphatidylinositols, phosphatidylserines, and sphingomyelins, as well as a small amount of free cholesterol and phosphatidic acids [163]. Except during periods of lipophagy, this assortment of lipid molecules is likely to go undetected by metabolomic investigations of plasma/serum in MASLD.

Nevertheless, a collection of phospholipids has been reported to be prominent in human plasma/serum in MASLD metabolomics investigations, including PA(13:0/17:1), PA(20:3/20:5), PC(14:1:22:6), PE(14:0/14:0), PE(18:0/22:6), PE(18:3/20:5), PE(18:4/18:4), PE(20:4/20:4), LPS(21:0), LPE(22:1) [137], LPC(26:0), LPC(28:0), PC(24:0), PC(36:2), PC(40:6) [138], LPE(17:0), LPC(14:0), LPC(18:0), LPC(18:3), LPC(20:3) [142], PE(18:0/22:6), PE(16:0/22:6) [148] in adults and LPE(20:0) [157], LPS(22:2), PE(14:0/15:0), PC(16:0/17:2), and LPE(16:0) in children [158]. These phospholipids are mainly phosphatidylcholines (PC) and phosphatidylethanolamines (PE), which are interchangeable, and their relative concentrations are believed to be essential for the health of the liver. Phosphatidylethanolamine *N*-methyltransferase (PEMT; EC 2.1.1.17) is an enzyme that transfers three methyl groups from *S*-adenosyl-L-methionine to convert ethanolamine to choline in PE. This enzyme is crucial for the production of PC and in the formation of lipid droplets [164]. The role of PEMT in obesity is well established both in clinical and mouse model studies [165]. *Pemt^−/−^* mice fed a HFD were protected against diet-induced obesity and insulin resistance but developed MASLD associated with a decreased PC:PE ratio. Several human mutations in *PEMT* are known and the loss of function mutation V175M (G523A) was reported to occur in 70% of NAFLD patients but only 40% of controls [166]. The profile of circulating phospholipids together with the underlying *PEMT* genetic polymorphism will contribute to the pathogenesis of MASLD.

Circulating sphingolipids may also be altered in MASLD with sphinganine and sphingosine elevated [148] with no reports of increased ceramide concentrations. Bile acids have also been reported to increase in the plasma/serum of fatty liver disease patients, with GC, TC, TCDC, TDC, and GLC elevated in ALD [127], TC [113], and GUDC 3-sulfate [148] in NAFLD.

In addition to lipids, the other major circulating metabolite group enhanced in relation to MASLD is the amino acids. Reports include enhanced tyrosine, glutamate, lysine, isoleucine [113], glutamate, isoleucine, leucine, valine, tyrosine [120], arginine, alanine, leucine, phenylalanine, tyrosine, valine, ornithine, proline [125], methionine sulfoxide, cystine [127], glutamate, tyrosine [138], glutamate [148], alanine, isoleucine, leucine, valine, tyrosine [150], methionine sulfoxide, valine [152], serine, leucine, isoleucine, tryptophan [157] in adults, tyrosine, and glutamate in adolescents [118] and phenylalanine, tyrosine, proline, alanine, arginine, leucine, and ornithine in children [151]. Both essential and nonessential amino acids are prominent in the circulation in MASLD, approximately 20% [167]. The number of reports of the nonessential amino acids were tyrosine (7), glutamate (5), alanine (3), arginine (2), ornithine (2), proline (2), cystine (1), and serine (1) and for the essential amino acids were leucine (5), isoleucine (4), valine (4), phenylalanine (2), methionine (sulfoxide) (2), lysine (1), and tryptophan (1). The two remaining essential amino acids, threonine and histidine, were not found to be elevated in MASLD. Furthermore, several dipeptides were elevated, including glutamylvaline, glutamylleucine, glutamylphenylalanine, glutamyltyrosine [113], glutamylvaline, glutamylisoleucine, and glutamylleucine [120]. Interestingly, these are all glutamate dipeptides of either branched-chain or aromatic amino acids.

The three reported human urinary metabolomic studies, one in adults [122] and two in children [93,128], and one with feces [138] do not further add to our understanding of the metabolic perturbations in MASLD. Regarding metabolomic investigations of the human liver, the single report [121] showed increased hepatic bile acids DCA and TCA and compared these findings to a rat liver model for NAFLD, which showed elevated CA and DCA, together with the amino acids citrulline, lysine, serine, and threonine. These authors concluded that “the metabolomics results indicate important differences between humans and rodents in the biochemical pathogenesis of the disease.”. A more detailed analysis of the metabolomic investigations of animals with fatty liver may or may not substantiate this statement.

#### 2.3.2. Metabolomics of MASLD in Experimental Animals

Table 2 contains data for 23 MASLD metabolomic studies conducted in experimental animals, 12 in mice, 6 in rats, 1 in each hamsters, cows and geese, and 2 species of fish. Two mouse urinary metabolomics studies of ALD revealed the ethanol metabolites ethyl sulfate and ethyl-β-D-glucuronide, together with 4-hydroxyphenylacetic acid and its sulfate in *Ppara*^+/+^ mice, but phenyllactate and indole-3-lactate in susceptible *Ppara*^−/−^ mice. The authors interpreted their findings as indicating that consumption of NAD^+^ for the oxidation of alcohol along with concomitant impairment of NAD^+^ biosynthesis led to a marked shift in the redox balance and an increase in the NADH/NAD^+^ ratio. This results in significant impairment of fatty acid β-oxidation in *Ppara*^−/−^ mice leading to steatosis. Additionally, tryptophan and phenylalanine lead to indole-3-pyruvate and phenylpyruvate, respectively, which are reduced to their corresponding lactates due to the excess of NADH [114,115]. Indole-3-lactate and phenyllactate would appear to be potential biomarkers for the development of ALD in humans, although this proposition has yet to be tested. Another ALD study that fed an ethanol diet to mice [116] produced elevated levels of fatty acids in the liver, including FA(2:0), FA(6:0), FA(12:0), FA(14:0), FA(16:1), and FA(20:3), underlining the point made above that the paucity of NAD^+^ led to a failure of fatty acid β-oxidation. A further ALD study feeding ethanol to mice examined the liver and found elevated bile acids TC, GC, THDC, TDC, and 7-keto-DC [145].

In a HFD mouse study of NAFLD, various metabolites were elevated in serum, including the amino acids methionine and tryptophan and the leucine degradation product ketoleucine (4-methyl-2-oxopentanoic acid) [117]. In a different approach to developing fatty liver in mice, *Mat1a*^−/−^ mice that had chronically low levels of hepatic *S*-adenosylmethionine and spontaneously developed steatohepatitis, underwent metabolomic examination of their livers together with controls. The amino acids methionine, serine, and threonine were elevated in the liver [168]. In a further investigation, mice were fed a high-fat, high-cholesterol, cholate diet for three weeks and consistently developed a hepatic pathology similar to NAFLD without changes to body weight. Their livers contained upregulated free cholesterol and multiple cholesteryl esters plus cholate, while their plasma contained the same cholesteryl esters plus cholate and deoxycholate [123]. Another HFD mouse study reported raised serum fatty acids FA(16:0), FA(18:0), FA(18:1), and FA(20:4) [144] and another found a constellation of noteworthy metabolites prominent in serum, including the two lipoamino acids *N*-palmitoylarginine and *N*-arachidonoylarginine [153]. The best known lipoamino acid is arachidonoylglycine, which is closely related to the endocannabinoid anandamide [169], which we have reported elevated in the plasma of hepatitis C virus-positive patients together with the lipoamino acid *N*-arachidonoyltaurine [39]. Lipoamino acids are reviewed in [170]. In a final mouse study, NAFLD was generated in lean mice with a methionine-choline-deficient diet and serum showed two preeminent phospholipids, PE(22:4/19:0) and PS(O-20:0/18:1), together with the unusual metabolites 2-hydroxypyridine and β-alanylhistamine [159]. 2-Hydroxypyridine tautomerizes to 2-pyridone, which is the preferred form in aqueous solution [171]. It is a principal metabolite of the phosphodiesterase 3 inhibitor adibendan in rats, rabbits, dogs and humans [172], but hitherto has not been reported as an endogenous metabolite. β-Alanylhistamine, also known as carcinine, is the inactivated form of the neurotransmitter histamine in the eye of *Drosophila melanogaster* [173], but there is scant evidence that it is an endogenous mammalian metabolite.

In summary, the mouse metabolomic investigations provided information about the underlying mechanisms of ALD. However, regarding NAFLD, a few clues were found, for example, elevated fatty acids suggestive of downregulation of β-oxidation, and no PC, only PE and PS, indicative of a reduced PC:PE ratio associated with MASLD (see above). The most surprising mouse discoveries concerned the appearance of circulating lipoamino acids, 2-pyridone, and carcinine during the development of NAFLD. These unfamiliar findings require confirmation.

Rats fed a HFD to develop NAFLD showed the very-long-chain highly unsaturated fatty acid octacosaoctaenoic acid (FA(28:8)) in their serum, together with a single cholesteryl ester (CE(12:0)) and the phosphatidyglycerol PG(14:0/18:1) [119]. A second HFD NAFLD rat investigation yielded the unexpected finding of 12(*R*)-hydroxyeicosatetraenoic acid (12(*R*)-HETE), a 12R-lipoxygenase metabolite of arachidonic acid, in serum. The enzyme ALOX12B is expressed primarily in the epithelial cells of the skin [174]. ALOX12 (EC 1.13.11.31), which produces 12(*S*)-HETE, is expressed predominantly in platelets and skin [175]. This production of 12(*R*)-HETE by HFD in rats was therefore unanticipated. Additional findings from this rat study included enhanced serum concentrations of the amino acids leucine, valine, isoleucine, proline, arginine, and tryptophan [129], consistent with human MASLD. Another rat study employed a methionine-choline-deficient diet to generate NAFLD, and analysis of hepatic metabolites found raised cholate and deoxycholate together with the amino acids citrulline, lysine, serine, and threonine [121]. Among the hepatic metabolites in another rat NAFLD study was the bile acid TCDC 3-sulfate [134]. A further rat ALD investigation reported elevated serum amino acids β-alanine, alanine, arginine, serine, tyrosine, and ornithine [135]. Other rat NAFLD studies reported elevated serum amino acids, energy metabolites, fatty acids, and bile acids [140,146,155].

Hamster liver with NAFLD revealed a number of enhanced metabolites including six LE and LPE, but only one LPC [143], consistent with a low hepatic PC:PE ratio due to attenuated PEMT activity, as observed in human MASLD (see above). In bovine NAFLD, analysis of cow serum, urine, and feces found only elevated fatty acids [147]. Overfed geese with NAFLD displayed unusual hepatic metabolites, for example, 3α, 7α, 12α-trihydroxycoprostane, otherwise known as 5β-cholestane-3α, 7α, 12α-triol, an intermediate in the synthesis of bile acids. Also enhanced in goose liver was the unsaturated aliphatic hydrocarbon squalene, which is a biochemical precursor for the steroid family. It has long been known that chick liver and kidneys can convert mevalonate into squalene, lanosterol, and cholesterol [176]. The finding of squalene and 3α, 7α, 12α-trihydroxycoprostane in NAFLD goose liver suggests attempts by the animal to synthesize bile acids de novo via cholesterol [177]. Investigations on two species of fish are included in Table 2. For blunt snout bream (*Megalobrama amblycephala*), a hand-fed, high-carbohydrate diet generated NAFLD and their serum was found to have prominent concentrations of glucose, succinate, and tyrosine [124]. These first two metabolites are unsurprising given the diet, but tyrosine is a common finding in mammalian species with MASLD (see above). Zebrafish larvae were exposed to ethanol and sections were subjected to whole-body mass spectrometry imaging by MALDI-MSI. Glutamate, taurine, malate, acylcarnitine C2, LPC(16:0), and PC(34:1) were all elevated relative to control zebrafish larvae [156], results consistent with the mammalian data above, including human outcomes.

#### 2.3.3. The Metabolomic Hallmarks of MASLD Preeminent in the Circulation

1. Energy metabolites
2. Essential amino acids
3. Nonessential amino acids
4. Fatty acids
5. Bile acids

These hallmarks are derived from the interface between 26 clinical and 24 experimental investigations. As shown in Table 2, the metabolomes of experimental animals that were analyzed included mice, rats, hamsters, cows, geese, and two species of fish.

#### 2.3.4. Comparison of the Obesity and MASLD Hallmarks

**Figure 2:**
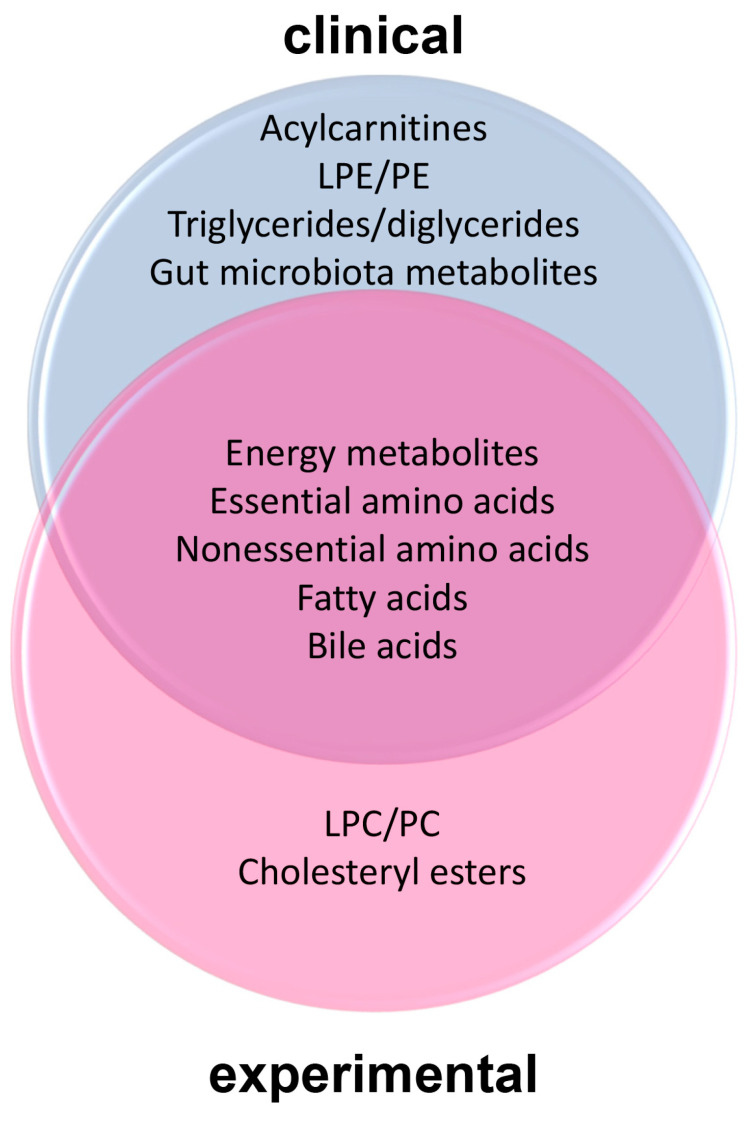
Venn diagram showing the shared elevated MASLD biomarker groups between clinical and experimental studies.

To examine if metabolomic features of obesity are drivers of MASLD, a comparative examination of the metabolomic hallmarks of the two diseases would be helpful. First, the three hallmarks shared between the two diseases were energy metabolites, essential amino acids, and nonessential amino acids. The unique metabolomic hallmarks for obesity were LPC, acylcarnitines, and gut microbiota metabolites (details in Table 1) and for MASLD the unique metabolomic hallmarks were fatty acids and bile acids (details in Table 2). Phosphatidylcholine (PC) is essential for the health of the liver [178]. In all nucleated mammalian cells, PC is synthesized by the CDP-choline pathway, also called the Kennedy pathway. However, in liver, ~30% PC is biosynthesized by an alternative pathway with the conversion of phosphatidylethanolamine (PE) to PC by PE-methyltransferase (PEMT). Furthermore, PC may be supplied by the reacylation of LPC [179,180]. A diminished molar ratio of PC:PE, as discussed above, appears to be a driver of MASLD. It is therefore interesting that the only phospholipids that appear in the hallmarks are LPC in the obesity hallmarks (see Figure 1). LPC/PC and LPE/PE appeared in the experimental and clinical sectors, respectively, of the MASLD Venn diagram (Figure 2), but not in both. Perhaps attenuated PEMT activity drives fatty liver development in humans but not in the animal models studied.

Two metabolomic hallmarks of MASLD shared first place. The first was upregulated energy metabolites. Examples included glycerol 3-phosphate and mannose in mouse serum [117], glucose and succinate in blunt snout bream serum [124], 2-hydroxyglutarate and glutaconate in mouse serum [131], 3-phosphoglycerate and glutarate in goose liver [133], fumarate, 2-oxoglutarate, fructose, mannose, glyceraldehyde, citrate, and glutamine in rat serum [140,155], glucose and gluconolactone in human urine [93], galactose, galactitol, and mannose in human serum, 2-oxoglutarate, pyruvate, and ribitol in human serum [148], pantothenate, citrate, citramalate, glutamine, glycerate, and ribose in human serum [149], propionate, formate, valerate, and α-methylbutyrate in human plasma [154]. These metabolites in both experimental and clinical settings are, in general, substrates and not products of energy metabolic reactions, suggesting that energy production in the liver is disrupted in MASLD.

The other metabolomic hallmark sharing first place was essential amino acids, which appeared in 6/24 experimental and 9/26 clinical investigations. The nine essential amino acids that cannot be synthesized in the human body are histidine, isoleucine, leucine, lysine, methionine, phenylalanine, threonine, tryptophan, and valine [181]. These appear in the experimental investigations as follows, isoleucine (2), leucine (2), lysine (2), tryptophan (4), threonine (2), and valine (2), and in the clinical studies as follows, isoleucine (4), leucine (4), lysine (1), methionine (1), phenylalanine (4), tryptophan (1), and valine (3). Therefore, 8/9 essential amino acids comprise the essential amino acid metabolomic hallmark of MASLD. Notably absent was the basic essential amino acid histidine.

The nonessential amino acids, for which we do not rely on dietary intake, comprise alanine, arginine, asparagine, aspartate, cysteine, glutamate, glutamine, glycine, proline, serine, and tyrosine. Nonessential amino acids were elevated in 4/24 experimental and 11/26 clinical investigations (Table 2). In the clinical studies, tyrosine (7) and glutamate (5) were the most prevalent, with proline (2), alanine (2), arginine (2), cystine/cysteine (2), serine (1), glutamine (1), and arginine (1). In the experimental studies, there were fewer reports of elevated nonessential amino acids with serine (3), alanine (2), tyrosine (2), arginine (1), and cysteine (1). A recent report suggested that the nonessential amino acid transporter SLC7A11 played a role in MASLD from loss-of-function and gain-of-function genetic models. Specifically, SLC7A11 deficiency accelerated MASLD progression via a classic cystine/cysteine deficiency-induced ferroptosis, while serine deficiency and a resulting impairment in de novo cysteine production were attributed to ferroptosis-induced MASLD progression in mice overexpressing hepatic SLC7A11 [182]. Nonessential amino acids may therefore be closely related to MASLD pathobiology. One clear example is tyrosine. A large biobank study that included 359 patients with NAFLD reported a strong association between NAFLD and blood tyrosine concentrations, but no other metabolite of the 123 measured. In an additional proof of concept study on bariatric surgery patients, blood tyrosine levels were higher in patients with NAFLD than without [183]. For a comprehensive review of amino acids in NAFLD, see [184].

The concentration of free fatty acids was raised in the circulation in 5/24 experimental and 3/26 clinical studies. An increasing body of evidence highlights perturbations in hepatic mitochondrial metabolism as a major contributor to the progression of NAFLD to NASH and fibrosis [185]. Mitochondrial fatty acid β-oxidation produces acetyl-CoA, which can be used in the citric acid cycle or diverted to ketogenesis, producing the ketone bodies acetoacetate, 3-hydroxybutyrate and acetone. It is believed that in MASLD, the combination of a high intrahepatic fatty acid content and insulin resistance may predispose patients to increased ketogenesis by providing more substrate for ketone body production [186]. Therefore, 3-hydroxybutyrate in particular has been employed as a surrogate measure of mitochondrial β-oxidation [185]. Both 3-hydroxybutyrate [185] and total ketone bodies [186] have been reported to be related to NAFLD and its severity. Interestingly, 3-hydroxybutyrate was a metabolomic finding in obesity (Table 1) in several studies involving obese mice [71], rats [87], and children [89], but was not reported in the metabolomic investigations of MASLD (Table 2). It is therefore likely that energy metabolites in obesity, other than 3-hydroxybutyrate, are involved in the pathogenesis of MASLD. A detailed lipidomic investigation of obese patients with and without MASLD, undergoing bariatric surgery, has recently been reported, in which hepatic levels of both odd-chain and even-chain fatty acids did not differ between these two groups, but both n-6 and n-3 polyunsaturated fatty acids were decreased in the livers of MASLD patients. FA(18:0) was statistically significantly lower in MASLD livers, while FA(18:1) was correspondingly higher. The elongation index (FA(18:0)/FA(16:0)) was lower in MASLD, but the desaturation index (FA(18:1)/FA(18:0)) was higher. De novo fatty acid synthesis (*FASN* mRNA by qPCR) was greater in MASLD liver than non-MASLD liver. The authors concluded that alterations in hepatic fatty acid levels in MASLD patients were due to enhancement of de novo lipogenesis in the liver [187].

### 2.4. Key Unanswered Questions and Potential Future Directions

Our review of the metabolomic hallmarks of obesity and MASLD reveal a number of unanswered questions, for example, the precise metabolic role of the gut microbiota in the pathogenesis of both obesity and MASLD. Recent work on obesity resolution after metabolic and bariatric surgery (MBS) has largely concentrated on the effects of MBS on gut microbiota composition [188,189]. Increases in several families and genera from the phylum Proteobacteria and Bacteroidetes, the family Streptococcaceae, the species *Akkermansia muciniphila*, and *Streptococcus salivarius* and a decrease in the phylum Firmicutes and the family Bifidobacteriaceae were reported [188]. However, little attention has been given to the metabolites produced by the gut microbiota as players in both obesity and MASLD. We have commented that the panoply of metabolites that can be produced by the microbiota, including ethanol, secondary bile acids, trimethylamine, indole, quinolone, phenazine, and their derivatives and the quorum sensor acyl homoserine lactones that all may contribute to liver disease have yet to be fully investigated [190]. As diet is a key factor in the pathogenesis of obesity and MASLD, it is hardly surprising that the gut microbiota can be affected by diet. We have reported that adding just one extra component to the human diet, in this case grapes, can significantly affect the gut microbiota composition following two weeks of grape consumption, taxonomic abundance was altered with decreased *Holdemania* spp. and increased *Streptococcus thermophiles* [191]. Addition of grape powder to a high-fat diet in mice reduced MASLD occurrence and improved longevity [192]. Analysis of the hepatic and urinary metabolomes of these mice revealed that gut microbiota metabolites 4-hydroxyphenylacetic acid, 5-hydroxyindole, glyceric acid, gluconic acid, and *myo*-inositol were attenuated when grapes were added to the standard diet but the gut microbiota metabolites gluconic acid, *scyllo*-inositol, mannitol, xylitol, 5-hydroxyindole, and 2-deoxyribonic acid were increased in urine when grapes were added to the high-fat diet [36]. To date, the precise microbial origins of each of these metabolites are unknown. These dietary effects on both humans and mice encapsulate a key unanswered question regarding the metabolic flux of the gut microbiota in relation to both obesity and MASLD. An important future research direction should be to catalog the exact microbial source of each urinary metabolite, as we have previously discussed [190].

Another key unanswered question is how metabolomics could contribute to the mechanistic understanding of the progression of MASLD to MASH and HCC. It was recently reported in humans with MASH, that liver injury correlated positively with ketogenesis and total fat oxidation, but not with turnover of the tricarboxylic acid cycle. This investigation utilized NMR spectroscopy, UPLC-MS, and GC-MS and performed stable-isotope tracing and formal flux modeling to quantify hepatic oxidative fluxes in humans across the spectrum of MASLD–MASH, and in mouse models of impaired ketogenesis [193]. It has been stated that the complexity of the mechanisms underlying MASLD progression remains a significant challenge for the development of effective therapeutics. These authors deleted miR-33 in the liver and found that fatty acid synthesis was attenuated, and mitochondrial fatty acid oxidation was increased, reducing the lipid burden on the liver. They suggested that suppressing hepatic miR-33 may be an effective therapeutic approach to temper the development of MASLD, MASH, and HCC in obesity [194]. In another study, expression of hepatic ornithine decarboxylase (ODC1) and therefore the production of putrescine was correlated with progression of MASLD [111]. A comprehensive metabolomic and lipidomic analysis of MASLD progression is, however, lacking and represents a potential future research direction.

Finally, our review demonstrates that the metabolic patterns of obesity in children, adolescents, and adults are not the same (Table 1; Section 2.2.2). Why this is the case is a key unanswered question. Are these differences due to hormones, diet, or some other ontogenetic factor? Biro and Wien stated in 2010: “The expression of genes favoring the storage of excess calories as fat, which have been selected for over many millennia and are relatively static, has become maladaptive in a rapidly changing environment that minimizes opportunities for energy expenditure and maximizes opportunities for energy intake” [195]. Children and adolescents have less control over their food intake and exercise compared to adults. This may be a contributing factor to obesity in early life. Nevertheless, our review shows that the limited metabolomic data suggests metabolic differences between obese children and obese adults. Is this due to ontogeny of the liver? Ontogeny of human hepatic enzymes has been addressed in the laboratory, including cytochromes P450 [196], aldehyde oxidase [197], and UDP-glucuronosyltransferases [198]. However, while these insights clarify the disposition of drugs in children compared to adults, sparce data are available on the ontogeny of lipid anabolism and catabolism. To understand better the metabolic differences between obese children and obese adults, future research should address these issues.

## 3. Conclusions

Metabolomics investigations in experimental animals and in clinical studies revealed a plethora of elevated metabolites in the circulation both in obesity and MASLD. Detailed analysis of these metabolite patterns yielded six metabolomic hallmarks for obesity: lysophosphatidylcholines, acylcarnitines, essential amino acids, nonessential amino acids, energy metabolites, and gut microbiota metabolites. Similarly, five metabolomic hallmarks of MASLD were derived: energy metabolites, essential amino acids, nonessential amino acids, fatty acids, and bile acids. These hallmarks represent a distillation of the metabolic character of these two diseases. These hallmarks also guide our understanding of how obesity may lead to MASLD.

## Figures and Tables

**Table 1:** Metabolomic studies conducted on obesity listed in chronological order.

Species	Pathology	Tissues Studied	Findings	Reference
rat	Zucker obese rat	liver, blood	methionine (X50), betaine (X4) ↓ in obese rat liver	[66]
rat	Zucker obese rat	plasma	LPC(16:0), LPC(18:1), LPC(18:0) ↑ in obese rat plasma	[67]
mouse	obese on high-fat diet (HFD)	liver, serum	arginine, tyrosine, pipecolic acid, benzoic acid, pantothenic acid, uric acid, phenylpyruvic acid, phenylacetamide, serotonin, L-carnitine, stearoylcarnitine, PCs, and 3 LPCs with C17:0, C18:0, and C18:3 ↑ in serum by HFD. Four acyl-carnitines (with C14:0, C16:1, C18:0, C18:1, and C18:2), 11 LPCs (with C14:0, C15:0, C16:0, C16:1, C17:1, C18:1, and C18:2, C19:0, C20:1, and C20:4), and two LPEs (with C18:2 and C20:4) ↓ in serum by HFD. 7-ketodeoxycholic acid, pantothenic acid, PCs and LPCs (with C20:4 and C22:6) ↑ in liver by HFD. valine, betaine, L-carnitine, 3-methylgutarylcarnitine, and LPCs (with C14:0, C16:0, C16:1, C18:0, and C18:3) ↓ in liver by HFD.	[68]
mouse	obese on HFD	serum	glucose ↑ in HF serum vs. LF isobutyrate, TMAO, creatine, valine, 3-methyl-2-oxovalerate, phenylalanine, isoleucine, leucine, taurine, glycine, *O*-acetylcarnitine, choline, glutamate, lactate, tyrosine, methionine, acetate ↓ in HF serum vs. LF	[69]
rat	ovariectomized (OVX) obese	serum	cholesterol, glycerol, glucose, arachidonic acid, glutamic acid, glycine, and cystine ↑ in OVX serum alanine ↓ in OVX	[70]
mouse	obese on HFD	serum	3-hydroxybutyrate, glutamine, 2-hydroxybutyrate, tyrosine, citrulline, glucose ↑ in HF serum vs. chow glutamate, fumarate, choline, urea ↓ in HF serum vs. chow	[71]
human	healthy obese	serum	glycine, glutamine, PC(42:0) ↑ in healthy obese vs. healthy lean PC(32:0), PC(31:1), PC(40:5) ↓ in healthy obese vs. healthy lean	[72]
human	obese adolescents	plasma	carnitine(10:0), histidine, serine ↓ in obese adolescents	[73]
human	obese PCOS, obese non-PCOS, non-obese PCOS, non-obese non-PCOS	plasma	linoleic acid, oleic acid, glycerol ↑ in obese PCOS palmitoleic acid, oleic acid, citramalic acid, phenylalanine, gluconic acid lactone in all obese ↑ glycine ↓ in all obese	[74]
rat	HFD, LFD, cafeteria diet (CAF)	serum, liver, muscle, adipose	myristic acid (14:0), palmitoleic acid (16:1), palmitic acid (16:0), α-linolenic acid (18:3), linoleic acid (18:2), oleic acid (18:1), stearic acid (18:0) ↑ in serum on CAF diet triglycerides ↑ in muscle on CAF diet C3, C4/C14, C5, C4-OH, C6, C8, C10, C12, C14, C16, C18:1, C18 acylcarnitines ↑ in muscle on CAF diet C10, C12, C18:1, C18 acylcarnitines ↑ in adipose on CAF diet	[75]
human	men and women with abdominal obesity	serum	correlated metabolites with android (A), gynoid (G), abdominal visceral (VAT), subcutaneous (SAT) fat. Triglycerides TG(54:1–3) correlated to VAT in women but TG(50:1–5), TG(55:1), PC(32:0) correlated to VAT in men.	[76]
mouse	ob/ob mice vs. B6 controls	urine	male mice: alanine, 5-aminolevulinate, guanidino-acetate, 2-hydroxybutyrate, 3-hydroxy-kynurenine, isopropanol, leucine, methionine, methylmalonate, *N*-acetyl aspartate, *N*-acetyl glutamate, 2-oxo-isocaproate, phenylalanine, threonine, tryptophan, tyrosine, valine, pyruvate, glycerol, creatine, creatine phosphate, creatinine, choline, dimethylamine, hippurate, 2-hydroxyisobutyrate, isobutyrate, methylamine, *p-*cresol, TMA, trigonelline, allantoin, , suberate, 2-hydroxyvalerate, nicotinamide *N*-oxide ↓ ob/ob vs. B6 controls female mice: alanine, 2-hydroxybutyrate, leucine, methionine, 2-oxo-isocaproate, phenylalanine, urea, acetate, taurine, creatine, creatinine, choline, methylamine, 2-hydroxyvalerate, suberate ↓ ob/ob vs. B6 controls acetoacetate, acetone, citrate, fumarate, 2-oxo-glutarate, succinate, TMA, 3-hydroxybutyrate ↑ ob/ob vs B6 controls male mice: arginine, lysine, ornithine, glucose, glycolate, pyruvate, creatine ↓ in ob/ob mice vs B6 controls acetoacetate, succinate, carnitine, TMAO, VLDL/LDL cholesterol ↑ in ob/ob vs B6 controls female mice: alanine, arginine, glycine, isoleucine, lysine, methionine, ornithine, serine, citrate, glycolate, lactate, creatine, choline, ethylene glycol ↓ in ob/ob vs B6 controls acetone, carnitine, VLDL/LDL cholesterol ↑ in ob/ob vs B6 controls	[77]
		serum	male mice: arginine, lysine, orni-thine, glucose, glycolate, pyruvate, creatine ↓ in ob/ob mice vs B6 con-trols acetoacetate, succinate, carnitine, TMAO, VLDL/LDL cholesterol ↑ in ob/ob vs B6 controls female mice: alanine, arginine, gly-cine, isoleucine, lysine, methionine, ornithine, serine, citrate, glycolate, lactate, creatine, choline, ethylene glycol ↓ in ob/ob vs B6 controls acetone, carnitine, VLDL/LDL cho-lesterol ↑ in ob/ob vs B6 controls	
human	childhood obese vs. lean	plasma	BCAA; valine, leucine, isoleucine, (and related intermediate metabolites) and androgens; dehydroepiandrosterone sulfate (and their metabolites) ↑ in obese than lean children	[78]
human	nondiabetic obese vs nondiabetic lean	serum	3-hydroxybutyric acid, lysine, glutamine, choline, proline, 3,7-dimethyluric acid, pantothenic acid, *myo*-inositol, threonine, leucine, sorbitol, glycerol, glucose, histidine ↑ in nondiabetic obese vs. nondiabetic lean	[79]
human	fasting obese vs lean young students	serum	alanine, valine, proline, creatine, asparagine, phenylalanine, leucine, isoleucine, FFA(14:0), FFA(16:0), FFA(16:1), FFA(18:0), FFA(18:1), FFA(18:2), FFA(18:3), FFA(20:2), FFA(20:5), FFA(22:4), FFA(22:5), FFA(22:6) ↑ in fasting obese vs fasting lean glutamate, glutamine, taurine ↓ in fasting obese vs fasting lean	[80]
human	obese vs lean males	skeletal muscle	2-oxoglutarate ↑ in obese skeletal muscle than lean skeletal muscle glycine, histidine, methionine, citrulline, C4, C8, C10, C10:1, C10:2, C12:1 acylcarnitines ↓ in obese skeletal muscle than lean skeletal muscle	[81]
		plasma	C3, C4, C10:1 acylcarnitines ↑ in obese plasma than lean plasma histidine ↓ in obese plasma than lean plasma	
human	obese vs normal weight men	serum	2-octenoylcarnitine, eicosadienoic acid, 12-hydroperoxyeicosatetraenoic acid, 4-hydroxyestrone sulfate, LPE [18:1(11Z)/0:0], thromboxane B2 and pyridinoline ↑ in obese men vitamin D3 glucuronide, 9,10-DHOME ↓ in obese men	[82]
human	obese vs nonobese Hispanic children	fasting plasma	alanine, creatine, glutamate, 3-methyl-2-oxobutyrate, α-hydroxyisovalerate, isoleucine, leucine, valine, lysine, α-hydroxybutyric acid, α-ketobutyric acid, 3-(4)-hydroxyphenyllactate, phenylalanine, tyrosine, *N*-(3-acetamidopropyl)pyrrolidin-2-one, *C*-glycosyl-tryptophan, kynurenate, kynurenine, tryptophan, ornithine, γ-glutamylglutamate, γ-glutamylleucine, γ-glutamylphenylalanine, γ-glutamyltyrosine, bradykinin, des-Arg9-bradykinin ↑ in obese Hispanic children asparagine, aspartate, pyroglutamine, glycine, *N*-acetylglycine, serine, histidine, citrulline ↓ in obese Hispanic children	[83]
human	metabolically healthy obese (MHO), lean healthy (LH) and metabolically unhealthy obese (MUO)	fasting serum	MUO < MHO < LH: asparagine, glutamine, cystine, serine LH < MHO ≈ MUO: FA(16:1) LH ≈ MUO < MHO: FA(20:4), FA(18:2) MUO < MHO ≈ LH: FA(18:3) Correlation with HOMA-IR: proline, leucine, FA(14:0), FA(16:0) Correlation with fasting glucose: creatine, proline, FA(14:0), FA(18:0), FA(14:1), FA(18:1), FA(18:2) Correlation with postprandial AUC glucose: FA(14:0), FA(16:0), FA(14:1) Correlation with postprandial AUC insulin: FA(16:0), isoleucine	[84]
human	MSG-treated obese mice	urine	2 months: 1-methylnicotinamide, 2-PY, 4-PY, citrate, succinate, acetate ↑ trigonelline, nicotinamide *N*-oxide, methylamine, creatine, *N*-isovalerylglycine, putrescine ↓ 6 months: 1-methylnicotinamide, 2-PY, 4-PY, phenacetylglycine, allantoin ↑ trigonelline, nicotinamide *N*-oxide, methylamine, *N*-isovalerylglycine, putrescine ↓ 9 months: 1-methylnicotinamide, 2-PY, 4-PY, phenacetylglycine, allantoin ↑ trigonelline, nicotinamide N-oxide, methylamine, N-isovalerylglycine, putrescine ↓	[85]
human	severely obese vs non-obese	serum	alanine, leucine, isoleucine, valine, tyrosine, phenylalanine, pyruvate, citrate, acetoacetate, glucose, VLDL1, formate, methanol, isopropanol ↑ glutamine, histidine ↓	[86]
rat	obese (HFD) vs lean (ND)	serum	lactate, alanine, 2-hydroxyisobutyrate, pyruvate, creatine/creatinine, glucose, acetate ↑ 3-hydroxybutyrate ↓	[87]
human	obese adolescents vs normal weight adolescents	urine	C3, C5, C8, C10, C12, C14 acylcarnitines, hydroxypropionyl carnitine, carnitine, aspartate, asymmetric dimethylarginine, putrescine ↑ carnitine, carnitine, aspartate, asymmetric dimethylarginine, putrescine ↑ glycine, serine, threonine, methionine, dopamine, isoleucine, arginine, ornithine, citrulline, carnosine, serotonin, C4 acylcarnitine, SM(16:0), SM(OH)(22:1), SM(24:1), PC aa 34:2, 38:6, 30:2, 34:4, 34:1, 38:5, 36:1 ↓	[88]
human	obese children with and without im-paired insulin sig-naling	serum	taurodeoxycholate, glycodeoxycholate, LPE(16:0), LPC(14:0), LPE(18:0), LPE(18:1), LPE(18:2), LPE(20:3), LPS(19:0), LPS(20:4), methylbutyrylcarnitine, threitol, piperidine, pyruvate, lactate, alanine, proline, valine, leucine, isoleucine, 2-ketoisocaproate, tryptophan, phenylalanine, tyrosine, arginine, aspartate ↑ acetylcarnitine, biliverdin, docosapentaenoate, docosahexaenoate, 3-hydroxybutyrate ↓	[89]
mouse	obese (HFD) vs lean (normal diet)	serum	glucose, glycine, alanine ↑ serine, isoleucine, valine, acetoacetate ↓	[90]
rat	obese (HFD) vs lean (normal diet)	urine	creatinine, cytosine, 7-methylhypoxanthine, glucosamine, indole-3-carboxylic acid glucuronide, indole-3-carboxylic acid, phenacetylglycine, 3-methoxyphenylpropanoic acid, 3-methyldioxyindole, indoxyl sulfate, *p*-cresol glucuronide, *p*-cresol sulfate, suberic acid ↑ hippuric acid, 4,6-dihydroxyquinoline, tyrosol, 4-pyridoxic acid, 2-phenylethanol glucuronide, 5-L-glutamyltaurine, cholic acid ↓	[91]
human	morbid obese vs non-obese	fasting serum	glutamate, 12 x ceramides ↑ glycine, LPC(16:0), LPC(17:0), LPC(18:0), LPC(18:1), LPC(18:2), LPE(18:0), LPE(18:1), LPE(18:2), PC(34:2), PC(34:3), PC(36:2), PC(36:3), PC(38:0), PC(38:5), PC(38:6), PC(40:6), PE(28:5), PE(36:0), PE(38:0), PE(38:1), PE(40:2), PE(40:3), PE(34:1), PE(34:2), PE(34:3), PE(36:2), PE(36:3), PE(38:2), PE(38:3), PE(38:6), PE(40:3), PE(40:5), PE(40:6), PS(38:4) ↓	[92]
human	obese vs normal weight children	urine	xylitol, phenylacetic acid ↓	[93]
human	obese gravidae vs normal weight gravidae	placenta	uracil, hypoxanthine, glucose-6-phosphate, 3-phosphoglycerate, glycerol, nicotinamide, tyrosine, phenylalanine, isoleucine, leucine, serine, palmitate ↑ lysine, taurine, aspartate, glutamine, inosine, guanosine, inositol, gluconate, docosahexaenoate, arachidonate, stearate ↓	[94]
human	metabolically unhealthy (MUHO) obese vs metabolically healthy (MHO)	plasma	MHO: BCAA, tyrosine, glutamate, PC(32:1), PC(38:3) ↑ acylcarnitine C18:2, LPC(18:0), LPC(18:1), LPC(18:2) ↓ MUHO: proline, PC(32:2), PC(34:2), C3 acylcarnitine, LPC(16:1) ↑ serine, asparagine, LPC(18:1), LPC(18:2), PC(34:3) ↓	[95]
human	obese and normal weight children	plasma	lactate ↑ glucose, cysteine, 2-oxoglutarate, citrate ↓	[96]
human	obese metabolic well and unwell vs lean well	plasma	alanine, α-aminoadipic acid, cystine, isoleucine, leucine, valine, phenylalanine, tyrosine, propionylcarnitine ↑ malonylcarnitine ↓	[97]
human	overweight/ obese men vs. normal weight men	serum	Phe-Phe, phenylalanine, tryptophan ↑ *p*-cresol, *p*-cresol sulfate, phenacetylglutamine, glutamine, sphingosine 1-phosphate ↓	[98]
		urine	glucuronic acid, uric acid, tetrahydrocortisone, deoxycortisol ↑ glutamine, phenacetylglutamine, indoxyl sulfate, *p*-cresol, *p*-cresol sulfate, phenylacetamide, 19-hydroxy-testosterone, tetrahydrodeoxycorticosterone ↓	
human	obese vs non-obese	fasting plasma	leucine, isoleucine, valine, alanine, glutamate, proline, tyrosine, LPC(16:1), PC(32:1), PC(32:2), PC(38:3) ↑ serine, asparagine, LPC(18:1), LPC(18:2), LPC(18:0), PC(34:3), PC(38:4), PC(40:6) ↓	[99]
human	obese vs non-obese	plasma	LPC(14:0), LPC(16:0), phenylalanine, tryptophan, tyrosine, isoleucine, leucine, valine, phenylacetamide, phenylpyruvic acid, uric acid, arginine ↑ LPC(18:1), LPC(18:2), LPC(20:4), LPC(20:5), acylcarnitines C8:0, C10:1, hypoxanthine ↓	[100]
human	lean vs normal weight obese (NWO) vs overweight obese (OWO)	plasma	OWO ≈ NWO > lean: linoleic acid, HPODE, HODE/EpOME, lysine, carnitine, proline ↑	[101]
human	healthy vs overweight vs stages 1,2,3 obesity	plasma	obese > overweight > healthy: steroidogenesis, androgen and estrogen metabolism, glycine and serine metabolism, homocysteine degradation, malate-aspartate shuttle, cysteine metabolism, beta-alanine metabolism, aspartate metabolism, taurine and hypotaurine metabolism, retinol metabolism, glutathione metabolism, glutamate metabolism, ammonia recycling, estrone metabolism, amino sugar metabolism, tryptophan metabolism, histidine metabolism, arginine, and proline metabolism ↑	[102]
human	link between sugar-sweetened beverages and obesity	plasma	5-hydroxylysine, glycine, γ-tocopherol/β-tocopherol, 2-oxoglutarate, N-acetylhistidine, butyrylcarnitine, cholesterol, 3-phenylpropionate, 9-hydroxystearate, 2-hydroxybutyrate/2-hydroxyisobutyrate, 3-hydroxybutyrylcarnitine, 3-hydroxyisobutyrate, 3-hydroxybutyrylcarnitine, 1,5-anhydroglucitol, erythronic acid, LPC(18:1), LPC(16:0), LPC(16:1), PC(18:2/18:2), PC(18:0/18:2), PC(18:2/18:3), LPE(18:2), LPI(18:1), LPE(18:1), PC(16:0/18:0), sphingomyelin(d18:1/20:0)/(d16:1/22:0), sphingomyelin(d18:2/14:0)/(d18:1/14:1) ↑	[103]
human	causes obesity effect of obesity both cause and effect of obesity	plasma	2-hydroxybutyrate, PC(34:4), acylcarnitine C6, PCE(18:1), cotinine ↑ valine, LPC(22:6), acylcarnitine C18 ↑ glycine, tyrosine ↑	[104]
human	obese women with PCOS vs obese women without PCOS	feces	taurocholate, FA(20:3), FA(20:4), FA(20:5), FA(22:4) FA(22:6), DHEA sulfate, 9,12,13-triHOME, pregnenolone sulfate, bilirubin ↑ testosterone, plastoquinol, xanthine, FA(24:1), thymine ↓	[105]

**Table 2:** Metabolomic studies conducted on MASLD detecting elevated metabolites listed in chronological order.

Species	Pathology	Tissues Studied	Findings	Reference
human	NAFLD	plasma	taurocholate, glutamylvaline, glutamylleucine, glutamylphenylalanine, glutamyltyrosine, FA(11:0), FA(18:3), acylcarnitines C0 and C4, mannose, lactate, glutamate, lysine, tyrosine, isoleucine ↑	[113]
mouse	ALD	urine	ethylsulfate, ethyl-β-D-glucuronide, 4-hydroxyphenylacetic acid, 4-hydroxyphenylacetic acid sulfate ↑, indole-3-lactic acid ↑ in PPARα-null mice only	[114]
mouse	ALD	urine	ethyl-β-D-glucuronide, *N*-acetylglycine ↑, phenyllactic acid, indole-3-lactic acid ↑ in PPARα-null mice only	[115]
mouse	ALD	liver	FA(2:0), FA(6:0), FA(12:0), FA(14:0), FA(16:1), FA(20:3), tyrosine, 2-aminobutyrate, glycolate, 3-pyridinol, hypoxanthine ↑	[116]
mouse	NAFLD	serum	methylhippurate, glycerol 3-phosphate, mannose, ketoleucine, 2-oxohexanoate, hydroxyphenyllactate, succinate, methionine, tryptophan ↑	[117]
human	NAFLD	plasma	tyrosine, glutamate, FA(8:0) ↑	[118]
rat	NAFLD	serum	FA(28:8), CE(12:0), PG(14:0/18:1) ↑	[119]
human	MASLD	serum	glutamate, isoleucine, valine, leucine, tyrosine, acylcarnitine C3, γ-glutamylvaline, γ-glutamylisoleucine, γ-glutamylleucine, urate, 3-methyl-2-oxovalerate, cyclo(leucylprolyl) ↑	[120]
human	NAFLD	liver	DCA, TCA ↑	[121]
rat	NAFLD	liver	CA, DCA, citrulline, lysine, serine, threonine ↑	
human	NAFLD	urine	acylcarnitines C0, C2, C10, 7-methylxanthine, 6β-hydroxy-testosterone ↑	[122]
human	NAFLD	urine	glucose, 1-methylhistidine, pseudouridine, glycolate, sebacate, glucono-1,4-lactone, 1-methylnicotinate, oxalate ↑	[93]
mouse	NAFLD nonobese	liver	free cholesterol, CE(16:1), CE(18:1), CE(18:2), CE(18:3). CE(20:1), CE(20:2), CE(20:3), CE(20:4), CE(22:5), CE(22:6), CA ↑	[123]
		plasma	CE(16:1), CE(18:1), CE(18:2), CE(18:3). CE(20:1), CE(20:2), CE(20:3), CE(22:5), CA, DCA ↑	
blunt snout bream	NAFLD	serum	glucose, succinate, tyrosine ↑	[124]
human	NAFLD	plasma	arginine, alanine, leucine, phenylalanine, tyrosine, valine, ornithine, proline, acylcarnitines C0, C10:2, C14:1 ↑	[125]
human	NAFLD	serum	TG(52:1), TG(53:1), TG(53:0), TG(58:2), TG(54:5) ↑	[126]
human	ALD	serum	glycocholate, taurocholate, taurochenodeoxycholate, glycodeoxycholate, taurodeoxycholate, glycolithocholate, *S*-methylmethionine, methionine sulfoxide, cystine, bilirubin (*Z,Z*), bilirubin (*E,E*), urobilinogen. 3β,17β-androstenediol monosulfate, 3β,17β-androstenediol disulfate, 5α--androstane-3β,17β-diol disulfate, isovalerate, 2-hydroxy-3-methylvalerate, α-hydroxyisovalerate, 2,3-dihydroxy-2-methylbutyrate ↑	[127]
human	NAFLD	urine	androgens (e.g., DHEA), glucocorticoids (e.g., tetrahydrocortisone), mineralocorticoids (e.g., corticosterone) ↑	[128]
rat	NAFLD	serum	12(*R*)-HETE, phosphatidylethanolamine, leucine, valine, isoleucine, proline, arginine, tryptophan, 2-hydroxycinnamic acid, *trans*-cinnamic acid ↑	[129]
human	NAFLD	plasma	acylcarntines C0, C3, C3DC, C4, C5, C5OH, C8:1, C10, C14OH, C14:1OH, C16:1, C16:2, C18, C18OH, C18:1, C18:2, C20, C20:4 ↑	[130]
mouse	NAFLD	serum	methylcysteine, tryptophan, tyrosine, alanine, *p*-cresol sulfate, 2-hydroxyglutarate, glutaconate, FA(22:4) ↑	[131]
	ALD	liver	FA(16:0), FA(18:2), FA(20:4), FA(22:5), xanthosine ↑	[132]
goose	NAFLD	liver	3-phosphoglycerate, glutarate, sphingosine, FA(24:0), 3α,7α,12α-trihydroxycoprostane, squalene, glutathione ↑	[133]
rat	NAFLD	liver	3-phosphoglycerate, taurochenodeoxycholate-3-sulfate, 4-hydroxy-6-eicosanone, 13-hydroxy-9-methoxy-10-oxo-11-octadecenate ↑	[134]
rat	ALD	serum	β-alanine, alanine, arginine, serine, tyrosine, ornithine ↑	[135]
human	NAFLD	serum	urate, galactose, galactitol, mannose, guanosine ↑	[136]
human	NAFLD	serum	glutaconate, homocitrulline, acylcarnitines C2, C3, LPE(22:1), PA(13:0/17:1), PA(20:3/20:5), PC(14:1:22:6), PE(14:0/14:0), PE(18:0/22:6), PE(18:3/20:5), PE(18:4/18:4), PE(20:4/20:4), LPS(21:0), MGDG(18:3/18:4) *N*-succinyldiaminopimelate, 20-COOH-leukotriene B4 ↑	[137]
human	MASLD	plasma	LPC(26:0), LPC(28:0), PC(24:0), PC(36:2), PC(40:6), glutamate, tyrosine ↑	[138]
		stool	cysteine, xanthine ↑	
human	NAFLD	serum	mannose, FA(18:0), FA(20:2), PI(18:0/20:4) ↑	[139]
rat	NAFLD	plasma	proline, fumarate, glucosylgalactosyl hydroxylysine, 3-methyl-1-hydroxybutyl-ThPP, 2-oxoglutarate, acetylphosphate, inosine triphosphate ↑	[140]
human	NAFLD	plasma	TG(54:0), TG(54:1), TG(53:0), TG(52:0), TG(50:0), TG(49:0), TG(48:0), TG(46:0), TG(45:1), TG(44:1), PC(32:1), LPE(16:1), CDCA, CA, LPE(20:4), LPE(22:5), androsterone sulfate ↑	[141]
human	NAFLD	serum	3-hydroxy-*cis*-5-tetradecenoylcarnitine, acylcarnitines C5, C8, C11-OH, C12-OH, C12-OHDC, LPE(17:0), LPC(14:0), LPC(18:0), LPC(18:3), LPC(20:3), MG(18:1), DG(18:1/18:2), DG(20:3/20:4), 25-hydroxyvitamin D3-26,23-lactol, deoxycholate 3-glucuronide, tuftsin, retinyl glucuronide, cortolone 3-glucuronide, tetrahydroaldosterone 3-glucuronide ↑	[142]
hamster	NAFLD	liver	glucosylceramide, PE(16:1/20:1), PE(16:1/20:2), PE(P-18:0/20:4), PC(18:0/18:2), DG(18:0/18:2), C16 sphingosine, *S*-(2-carboxypropyl)-cysteamine, tetrahydrodipicolinate, glycerol 3-phosphate, LPC(20:2), LPE(20:1), LPE(20:2), LPE(20:3) ↑	[143]
mouse	NAFLD	serum	FA(16:0), FA(18:0), FA(18:1), FA(20:4) ↑	[144]
mouse	ALD	liver	taurocholate, glycocholate, taurohyodeoxycholate, taurodeoxycholate, 7-keto-deoxycholate ↑	[145]
rat	NAFLD	serum	proline, lysine, tryptophan, citrulline, isoleucine, valine, arginine, leucine, sphingosine-1-phosphate, glycocholate, urate, stearate, palmitate, glycerylphosphorylethanolamine, TG(18:0/20:4/20:4), glycerol, 12(*R*)-HETE, galactose ↑	[146]
cow	NAFLD	feces urine serum	FA(22:0) ↑ FA(16:1) ↑ FA(17:0), FA(18:0), FA(19:0), FA(18:1,6*Z*) ↑	[147]
human	MAFLD	serum	1-carboxyethylisoleucine, 1-carboxyethyltyrosine, 1-carboxyethylphenylalanine, 2-oxoglutarate, acylcarnitines C4OH, C8OH, PE(18:0/22:6), PE(16:0/22:6), formiminoglutamate, glutamate, glycoursodeoxycholate 3-sulfate, pyruvate, 2-hydroxybutyrate/2-hydroxyisobutyrate, ribitol, sphinganine, sphingosine ↑	[148]
human	NAFLD	serum	pantothenate, hypoxanthine, citrate, citramalate, phenylalanine, glutamine, 1,4-butynediol, pyroglutamate, dehydroisoandroste-rone sulfate (DHEA-S), 5-androsten-3β,17β-diol-3-sulfate, glycerate, ribose, and 5α-pregnan-3α,17-diol-20-one 3-sulfate ↑	[149]
human	NAFLD	plasma	alanine, isoleucine, leucine, valine, tyrosine, lactate ↑	[150]
human	NAFLD	serum	phenylalanine, tyrosine, proline, alanine, arginine, leucine, ornithine, urate, carnitine, acylcarnitines C6, C8, C10, C10:2 ↑	[151]
human	ALD	serum	indolebutyrate, methionine sulfoxide, 3-ureidopropionate, *cis*-3,3-methyleneheptanoylglycine, retinol, valine ↑	[152]
mouse	NAFLD	serum	*N*-palmitoylarginine, sphingosine, arachidonoylarginine, LPC(20:2), LPC(20:3), PC(20:5-3-OH/2:0), LPI(20:4), Cer(d18:0/18:0)	[153]
human	MASLD	plasma	propionate, formate, valerate, α-methylbutyrate	[154]
rat	NAFLD	serum	FA(18:3), AMP, dihydrothymine, uracil, arabinonate, fructose, mannose, glyceraldehyde, dihydroorotate, citrate, glutamine, GS-SG, homocystate, β-alanine, TCA, DCA, GCA, GCDCA, PI(34:2), PI(38:5), FA(18:1, 12,13-di-OH), FA(20:1), 3-hydroxy-3-methylglutarate, glycerol 2-phosphate, LPE(18:1) ↑	[155]
zebrafish	ALD	whole body	glutamate, taurine, malate, acylcarnitine C2, LPC(16:0), PC(34:1)	[156]
human	MASLD	serum	serine, leucine, isoleucine, tryptophan, LPE(20:0) ↑	[157]
human	NAFLD	serum	sulfoacetate, gallate, pregnanetriol, LPS(22:2), FA(20:4), 1-lauroylglycerol, adenine, PE(14:0/15:0), PC(16:0/17:2), LPE(16:0) ↑	[158]
mouse	NAFLD	plasma	PE(22:4/19:0), PS(O-20:0/18:1), 2-hydroxypyridine, β-alanyl histamine ↑	[159]
human	NAFLD	saliva	aconitate, cholesterol ↑	[160]

## References

[1] Nasa P., Jain R., Juneja D. (2021). Delphi methodology in healthcare research: How to decide its appropriateness. World J. Methodol..

[2] Rinella M.E., Lazarus J.V., Ratziu V., Francque S.M., Sanyal A.J., Kanwal F., Romero D., Abdelmalek M.F., Anstee Q.M., Arab J.P. (2023). A multisociety Delphi consensus statement on new fatty liver disease nomenclature. J. Hepatol..

[3] Eslam M., Newsome P.N., Sarin S.K., Anstee Q.M., Targher G., Romero-Gomez M., Zelber-Sagi S., Wai-Sun Wong V., Dufour J.F., Schattenberg J.M. (2020). A new definition for metabolic dysfunction-associated fatty liver disease: An international expert consensus statement. J. Hepatol..

[4] Eslam M., Sanyal A.J., George J., International Consensus P. (2020). MAFLD: A Consensus-Driven Proposed Nomenclature for Metabolic Associated Fatty Liver Disease. Gastroenterology.

[5] Wang X., Zhang L., Dong B. (2024). Molecular mechanisms in MASLD/MASH-related HCC. Hepatology.

[6] Riazi K., Azhari H., Charette J.H., Underwood F.E., King J.A., Afshar E.E., Swain M.G., Congly S.E., Kaplan G.G., Shaheen A.A. (2022). The prevalence and incidence of NAFLD worldwide: A systematic review and meta-analysis. Lancet Gastroenterol. Hepatol..

[7] Miao L., Targher G., Byrne C.D., Cao Y.Y., Zheng M.H. (2024). Current status and future trends of the global burden of MASLD. Trends Endocrinol. Metab..

[8] Jarvis H., Craig D., Barker R., Spiers G., Stow D., Anstee Q.M., Hanratty B. (2020). Metabolic risk factors and incident advanced liver disease in non-alcoholic fatty liver disease (NAFLD): A systematic review and meta-analysis of population-based observational studies. PLoS Med..

[9] Younossi Z.M., Golabi P., de Avila L., Paik J.M., Srishord M., Fukui N., Qiu Y., Burns L., Afendy A., Nader F. (2019). The global epidemiology of NAFLD and NASH in patients with type 2 diabetes: A systematic review and meta-analysis. J. Hepatol..

[10] Yki-Jarvinen H. (2014). Non-alcoholic fatty liver disease as a cause and a consequence of metabolic syndrome. Lancet Diabetes Endocrinol..

[11] Wu X., Cheung C.K.Y., Ye D., Chakrabarti S., Mahajan H., Yan S., Song E., Yang W., Lee C.H., Lam K.S.L. (2022). Serum Thrombospondin-2 Levels Are Closely Associated With the Severity of Metabolic Syndrome and Metabolic Associated Fatty Liver Disease. J. Clin. Endocrinol. Metab..

[12] Nasereldin D.S., White L.J., Hodge D.O., Roberts L.R., Patel T., Antwi S.O. (2022). Association of metabolic health phenotypes, obesity, and hepatocellular carcinoma risk. Dig. Liver Dis..

[13] Alkhouri N., Almomani A., Le P., Payne J.Y., Asaad I., Polanco P., Leff P., Kumar P., Noureddin M. (2024). The prevalence of metabolic dysfunction-associated steatotic liver disease (MASLD)-related advanced fibrosis and cirrhosis in the United States population utilizing AGILE 3 + and AGILE 4 scores: Analysis of the NHANES 2017-2018 cycle. BMC Gastroenterol..

[14] Miyao M., Kotani H., Ishida T., Kawai C., Manabe S., Abiru H., Tamaki K. (2015). Pivotal role of liver sinusoidal endothelial cells in NAFLD/NASH progression. Lab. Investig..

[15] Adams J.C., Lawler J. (2011). The thrombospondins. Cold Spring Harb. Perspect. Biol..

[16] Hutchison A.L., Tavaglione F., Romeo S., Charlton M. (2023). Endocrine aspects of metabolic dysfunction-associated steatotic liver disease (MASLD): Beyond insulin resistance. J. Hepatol..

[17] De Chiara F., Heeboll S., Marrone G., Montoliu C., Hamilton-Dutoit S., Ferrandez A., Andreola F., Rombouts K., Gronbaek H., Felipo V. (2018). Urea cycle dysregulation in non-alcoholic fatty liver disease. J. Hepatol..

[18] Mercado-Gomez M., Goikoetxea-Usandizaga N., Kerbert A.J.C., Gracianteparaluceta L.U., Serrano-Macia M., Lachiondo-Ortega S., Rodriguez-Agudo R., Gil-Pitarch C., Simon J., Gonzalez-Recio I. (2024). The lipopolysaccharide-TLR4 axis regulates hepatic glutaminase 1 expression promoting liver ammonia build-up as steatotic liver disease progresses to steatohepatitis. Metabolism.

[19] Avila-Calderon E.D., Ruiz-Palma M.D.S., Aguilera-Arreola M.G., Velazquez-Guadarrama N., Ruiz E.A., Gomez-Lunar Z., Witonsky S., Contreras-Rodriguez A. (2021). Outer Membrane Vesicles of Gram-Negative Bacteria: An Outlook on Biogenesis. Front. Microbiol..

[20] Vaure C., Liu Y. (2014). A comparative review of toll-like receptor 4 expression and functionality in different animal species. Front. Immunol..

[21] Guo J., Friedman S.L. (2010). Toll-like receptor 4 signaling in liver injury and hepatic fibrogenesis. Fibrogenesis Tissue Repair..

[22] Gallage S., Ali A., Barragan Avila J.E., Seymen N., Ramadori P., Joerke V., Zizmare L., Aicher D., Gopalsamy I.K., Fong W. (2024). A 5:2 intermittent fasting regimen ameliorates NASH and fibrosis and blunts HCC development via hepatic PPARalpha and PCK1. Cell Metab..

[23] Beyoglu D., Popov Y.V., Idle J.R. (2024). The Metabolomic Footprint of Liver Fibrosis. Cells.

[24] Patterson A.D., Maurhofer O., Beyoglu D., Lanz C., Krausz K.W., Pabst T., Gonzalez F.J., Dufour J.F., Idle J.R. (2011). Aberrant lipid metabolism in hepatocellular carcinoma revealed by plasma metabolomics and lipid profiling. Cancer Res..

[25] Fahrner R., Beyoglu D., Beldi G., Idle J.R. (2012). Metabolomic markers for intestinal ischemia in a mouse model. J. Surg. Res..

[26] Beyoglu D., Imbeaud S., Maurhofer O., Bioulac-Sage P., Zucman-Rossi J., Dufour J.F., Idle J.R. (2013). Tissue metabolomics of hepatocellular carcinoma: Tumor energy metabolism and the role of transcriptomic classification. Hepatology.

[27] Beyoglu D., Krausz K.W., Martin J., Maurhofer O., Dorow J., Ceglarek U., Gonzalez F.J., Dufour J.F., Idle J.R. (2014). Disruption of tumor suppressor gene Hint1 leads to remodeling of the lipid metabolic phenotype of mouse liver. J. Lipid Res..

[28] Semmo N., Weber T., Idle J.R., Beyoglu D. (2015). Metabolomics reveals that aldose reductase activity due to AKR1B10 is upregulated in hepatitis C virus infection. J. Viral Hepat..

[29] Wang M., Keogh A., Treves S., Idle J.R., Beyoglu D. (2016). The metabolomic profile of gamma-irradiated human hepatoma and muscle cells reveals metabolic changes consistent with the Warburg effect. PeerJ.

[30] Simillion C., Semmo N., Idle J.R., Beyoglu D. (2017). Robust Regression Analysis of GCMS Data Reveals Differential Rewiring of Metabolic Networks in Hepatitis B and C Patients. Metabolites.

[31] Keogh A., Senkardes S., Idle J.R., Kucukguzel S.G., Beyoglu D. (2017). A Novel Anti-Hepatitis C Virus and Antiproliferative Agent Alters Metabolic Networks in HepG2 and Hep3B Cells. Metabolites.

[32] Patel D.P., Krausz K.W., Xie C., Beyoglu D., Gonzalez F.J., Idle J.R. (2017). Metabolic profiling by gas chromatography-mass spectrometry of energy metabolism in high-fat diet-fed obese mice. PLoS ONE.

[33] Golla S., Golla J.P., Krausz K.W., Manna S.K., Simillion C., Beyoglu D., Idle J.R., Gonzalez F.J. (2017). Metabolomic Analysis of Mice Exposed to Gamma Radiation Reveals a Systemic Understanding of Total-Body Exposure. Radiat. Res..

[34] Pabst T., Kortz L., Fiedler G.M., Ceglarek U., Idle J.R., Beyoglu D. (2017). The plasma lipidome in acute myeloid leukemia at diagnosis in relation to clinical disease features. BBA Clin..

[35] Idle J.R., Seipel K., Bacher U., Pabst T., Beyoglu D. (2020). (2R,3S)-Dihydroxybutanoic Acid Synthesis as a Novel Metabolic Function of Mutant Isocitrate Dehydrogenase 1 and 2 in Acute Myeloid Leukemia. Cancers.

[36] Beyoglu D., Park E.J., Quinones-Lombrana A., Dave A., Parande F., Pezzuto J.M., Idle J.R. (2022). Addition of grapes to both a standard and a high-fat Western pattern diet modifies hepatic and urinary metabolite profiles in the mouse. Food Funct..

[37] Beyoglu D., Simillion C., Storni F., De Gottardi A., Idle J.R. (2022). A Metabolomic Analysis of Cirrhotic Ascites. Molecules.

[38] Beyoglu D., Huang P., Skelton-Badlani D., Zong C., Popov Y.V., Idle J.R. (2023). Metabolic Hijacking of Hexose Metabolism to Ascorbate Synthesis Is the Unifying Biochemical Basis of Murine Liver Fibrosis. Cells.

[39] Beyoglu D., Schwalm S., Semmo N., Huwiler A., Idle J.R. (2023). Hepatitis C Virus Infection Upregulates Plasma Phosphosphingolipids and Endocannabinoids and Downregulates Lysophosphoinositols. Int. J. Mol. Sci..

[40] Hang D., Yang X., Lu J., Shen C., Dai J., Lu X., Jin G., Hu Z., Gu D., Ma H. (2022). Untargeted plasma metabolomics for risk prediction of hepatocellular carcinoma: A prospective study in two Chinese cohorts. Int. J. Cancer.

[41] Johnson C.H., Ivanisevic J., Siuzdak G. (2016). Metabolomics: Beyond biomarkers and towards mechanisms. Nat. Rev. Mol. Cell Biol..

[42] Benjamini Y., Hochberg Y. (1995). Controlling the False Discovery Rate: A Practical and Powerful Approach to Multiple Testing. J. R. Statist. Soc. B.

[43] Benjamini Y. (2010). Discovering the false discovery rate. J. R. Statist. Soc. B.

[44] Moran M. (2003). Arguments for rejecting the sequential Bonferroni in ecological studies. Oikos.

[45] Nakagawa S. (2004). A farewell to Bonferroni: The problems of low statistical power and publication bias. Behav. Ecol..

[46] Yun J.H., Kim J.M., Jeon H.J., Oh T., Choi H.J., Kim B.J. (2020). Metabolomics profiles associated with diabetic retinopathy in type 2 diabetes patients. PLoS ONE.

[47] Li L.J., Wang X., Chong Y.S., Chan J.K.Y., Tan K.H., Eriksson J.G., Huang Z., Rahman M.L., Cui L., Zhang C. (2023). Exploring preconception signatures of metabolites in mothers with gestational diabetes mellitus using a non-targeted approach. BMC Med..

[48] Yang M., Zhu C., Du L., Huang J., Lu J., Yang J., Tong Y., Zhu M., Song C., Shen C. (2023). A Metabolomic Signature of Obesity and Risk of Colorectal Cancer: Two Nested Case-Control Studies. Metabolites.

[49] Hu W., Wang W., Liao H., Bulloch G., Zhang X., Shang X., Huang Y., Hu Y., Yu H., Yang X. (2024). Metabolic profiling reveals circulating biomarkers associated with incident and prevalent Parkinson’s disease. NPJ Park. Dis..

[50] Harrell F.E. (2010). Regression Modeling Strategies: With Applications to Linear Models, Logistic Regression, and Survival Analysis.

[51] Castle A.L., Fiehn O., Kaddurah-Daouk R., Lindon J.C. (2006). Metabolomics Standards Workshop and the development of international standards for reporting metabolomics experimental results. Brief. Bioinform..

[52] Sumner L.W., Amberg A., Barrett D., Beale M.H., Beger R., Daykin C.A., Fan T.W., Fiehn O., Goodacre R., Griffin J.L. (2007). Proposed minimum reporting standards for chemical analysis Chemical Analysis Working Group (CAWG) Metabolomics Standards Initiative (MSI). Metabolomics.

[53] Members M.S.I.B., Sansone S.A., Fan T., Goodacre R., Griffin J.L., Hardy N.W., Kaddurah-Daouk R., Kristal B.S., Lindon J., Mendes P. (2007). The metabolomics standards initiative. Nat. Biotechnol..

[54] Spicer R.A., Salek R., Steinbeck C. (2017). A decade after the metabolomics standards initiative it’s time for a revision. Sci. Data.

[55] Alden N., Krishnan S., Porokhin V., Raju R., McElearney K., Gilbert A., Lee K. (2017). Biologically Consistent Annotation of Metabolomics Data. Anal. Chem..

[56] Woldemariam S., Dorner T.E., Wiesinger T., Stein K.V. (2023). Multi-omics approaches for precision obesity management: Potentials and limitations of omics in precision prevention, treatment and risk reduction of obesity. Wien. Klin. Wochenschr..

[57] Abraham A., Yaghootkar H. (2023). Identifying obesity subtypes: A review of studies utilising clinical biomarkers and genetic data. Diabet. Med..

[58] Ding J., Liu H., Zhang X., Zhao N., Peng Y., Shi J., Chen J., Chi X., Li L., Zhang M. (2024). Integrative multiomic analysis identifies distinct molecular subtypes of NAFLD in a Chinese population. Sci. Transl. Med..

[59] Beyoglu D., Idle J.R. (2020). Metabolomic and Lipidomic Biomarkers for Premalignant Liver Disease Diagnosis and Therapy. Metabolites.

[60] Mathe E.A., Patterson A.D., Haznadar M., Manna S.K., Krausz K.W., Bowman E.D., Shields P.G., Idle J.R., Smith P.B., Anami K. (2014). Noninvasive urinary metabolomic profiling identifies diagnostic and prognostic markers in lung cancer. Cancer Res..

[61] Kim H.Y. (2021). Recent advances in nonalcoholic fatty liver disease metabolomics. Clin. Mol. Hepatol..

[62] Reinson T., Buchanan R.M., Byrne C.D. (2023). Noninvasive serum biomarkers for liver fibrosis in NAFLD: Current and future. Clin. Mol. Hepatol..

[63] Yip T.C., Lyu F., Lin H., Li G., Yuen P.C., Wong V.W., Wong G.L. (2023). Non-invasive biomarkers for liver inflammation in non-alcoholic fatty liver disease: Present and future. Clin. Mol. Hepatol..

[64] Griffin J.L., Nicholls A.W. (2006). Metabolomics as a functional genomic tool for understanding lipid dysfunction in diabetes, obesity and related disorders. Pharmacogenomics.

[65] Gulston M.K., Titman C.M., Griffin J.L. (2007). Applications of metabolomics to understanding obesity in mouse and man. Biomark. Med..

[66] Serkova N.J., Jackman M., Brown J.L., Liu T., Hirose R., Roberts J.P., Maher J.J., Niemann C.U. (2006). Metabolic profiling of livers and blood from obese Zucker rats. J. Hepatol..

[67] Loftus N., Miseki K., Iida J., Gika H.G., Theodoridis G., Wilson I.D. (2008). Profiling and biomarker identification in plasma from different Zucker rat strains via high mass accuracy multistage mass spectrometric analysis using liquid chromatography/mass spectrometry with a quadrupole ion trap-time of flight mass spectrometer. Rapid Commun. Mass. Spectrom..

[68] Kim H.J., Kim J.H., Noh S., Hur H.J., Sung M.J., Hwang J.T., Park J.H., Yang H.J., Kim M.S., Kwon D.Y. (2011). Metabolomic analysis of livers and serum from high-fat diet induced obese mice. J. Proteome Res..

[69] Duggan G.E., Hittel D.S., Sensen C.W., Weljie A.M., Vogel H.J., Shearer J. (2011). Metabolomic response to exercise training in lean and diet-induced obese mice. J. Appl. Physiol. (1985).

[70] Ma B., Zhang Q., Wang G.J., A J.Y., Wu D., Liu Y., Cao B., Liu L.S., Hu Y.Y., Wang Y.L. (2011). GC-TOF/MS-based metabolomic profiling of estrogen deficiency-induced obesity in ovariectomized rats. Acta Pharmacol. Sin..

[71] Duggan G.E., Hittel D.S., Hughey C.C., Weljie A., Vogel H.J., Shearer J. (2011). Differentiating short- and long-term effects of diet in the obese mouse using (1) H-nuclear magnetic resonance metabolomics. Diabetes Obes. Metab..

[72] Oberbach A., Bluher M., Wirth H., Till H., Kovacs P., Kullnick Y., Schlichting N., Tomm J.M., Rolle-Kampczyk U., Murugaiyan J. (2011). Combined proteomic and metabolomic profiling of serum reveals association of the complement system with obesity and identifies novel markers of body fat mass changes. J. Proteome Res..

[73] Mihalik S.J., Michaliszyn S.F., de las Heras J., Bacha F., Lee S., Chace D.H., DeJesus V.R., Vockley J., Arslanian S.A. (2012). Metabolomic profiling of fatty acid and amino acid metabolism in youth with obesity and type 2 diabetes: Evidence for enhanced mitochondrial oxidation. Diabetes Care.

[74] Escobar-Morreale H.F., Samino S., Insenser M., Vinaixa M., Luque-Ramirez M., Lasuncion M.A., Correig X. (2012). Metabolic heterogeneity in polycystic ovary syndrome is determined by obesity: Plasma metabolomic approach using GC-MS. Clin. Chem..

[75] Sampey B.P., Freemerman A.J., Zhang J., Kuan P.F., Galanko J.A., O’Connell T.M., Ilkayeva O.R., Muehlbauer M.J., Stevens R.D., Newgard C.B. (2012). Metabolomic profiling reveals mitochondrial-derived lipid biomarkers that drive obesity-associated inflammation. PLoS ONE.

[76] Szymanska E., Bouwman J., Strassburg K., Vervoort J., Kangas A.J., Soininen P., Ala-Korpela M., Westerhuis J., van Duynhoven J.P., Mela D.J. (2012). Gender-dependent associations of metabolite profiles and body fat distribution in a healthy population with central obesity: Towards metabolomics diagnostics. OMICS.

[77] Won E.Y., Yoon M.K., Kim S.W., Jung Y., Bae H.W., Lee D., Park S.G., Lee C.H., Hwang G.S., Chi S.W. (2013). Gender-specific metabolomic profiling of obesity in leptin-deficient ob/ob mice by 1H NMR spectroscopy. PLoS ONE.

[78] Perng W., Gillman M.W., Fleisch A.F., Michalek R.D., Watkins S.M., Isganaitis E., Patti M.E., Oken E. (2014). Metabolomic profiles and childhood obesity. Obesity.

[79] Calvani R., Brasili E., Pratico G., Sciubba F., Roselli M., Finamore A., Marini F., Marzetti E., Miccheli A. (2014). Application of NMR-based metabolomics to the study of gut microbiota in obesity. J. Clin. Gastroenterol..

[80] Liu L., Feng R., Guo F., Li Y., Jiao J., Sun C. (2015). Targeted metabolomic analysis reveals the association between the postprandial change in palmitic acid, branched-chain amino acids and insulin resistance in young obese subjects. Diabetes Res. Clin. Pract..

[81] Baker P.R., Boyle K.E., Koves T.R., Ilkayeva O.R., Muoio D.M., Houmard J.A., Friedman J.E. (2015). Metabolomic analysis reveals altered skeletal muscle amino acid and fatty acid handling in obese humans. Obesity.

[82] Wang Y., Liu D., Li Y., Guo L., Cui Y., Zhang X., Li E. (2016). Metabolomic analysis of serum from obese adults with hyperlipemia by UHPLC-Q-TOF MS/MS. Biomed. Chromatogr..

[83] Butte N.F., Liu Y., Zakeri I.F., Mohney R.P., Mehta N., Voruganti V.S., Goring H., Cole S.A., Comuzzie A.G. (2015). Global metabolomic profiling targeting childhood obesity in the Hispanic population. Am. J. Clin. Nutr..

[84] Badoud F., Lam K.P., Perreault M., Zulyniak M.A., Britz-McKibbin P., Mutch D.M. (2015). Metabolomics Reveals Metabolically Healthy and Unhealthy Obese Individuals Differ in their Response to a Caloric Challenge. PLoS ONE.

[85] Pelantova H., Bartova S., Anyz J., Holubova M., Zelezna B., Maletinska L., Novak D., Lacinova Z., Sulc M., Haluzik M. (2016). Metabolomic profiling of urinary changes in mice with monosodium glutamate-induced obesity. Anal. Bioanal. Chem..

[86] Gralka E., Luchinat C., Tenori L., Ernst B., Thurnheer M., Schultes B. (2015). Metabolomic fingerprint of severe obesity is dynamically affected by bariatric surgery in a procedure-dependent manner. Am. J. Clin. Nutr..

[87] Gooda Sahib Jambocus N., Saari N., Ismail A., Khatib A., Mahomoodally M.F., Abdul Hamid A. (2016). An Investigation into the Antiobesity Effects of Morinda citrifolia L. Leaf Extract in High Fat Diet Induced Obese Rats Using a (1)H NMR Metabolomics Approach. J. Diabetes Res..

[88] Cho K., Moon J.S., Kang J.H., Jang H.B., Lee H.J., Park S.I., Yu K.S., Cho J.Y. (2017). Combined untargeted and targeted metabolomic profiling reveals urinary biomarkers for discriminating obese from normal-weight adolescents. Pediatr. Obes..

[89] Mastrangelo A., Martos-Moreno G.A., Garcia A., Barrios V., Ruperez F.J., Chowen J.A., Barbas C., Argente J. (2016). Insulin resistance in prepubertal obese children correlates with sex-dependent early onset metabolomic alterations. Int. J. Obes..

[90] Abdul Ghani Z.D., Husin J.M., Rashid A.H., Shaari K., Chik Z. (2016). Biochemical studies of Piper betle L leaf extract on obese treated animal using 1H-NMR-based metabolomic approach of blood serum samples. J. Ethnopharmacol..

[91] Men L., Pi Z., Zhou Y., Wei M., Liu Y., Song F., Liu Z. (2017). Urine metabolomics of high-fat diet induced obesity using UHPLC-Q-TOF-MS. J. Pharm. Biomed. Anal..

[92] Tulipani S., Palau-Rodriguez M., Minarro Alonso A., Cardona F., Marco-Ramell A., Zonja B., Lopez de Alda M., Munoz-Garach A., Sanchez-Pla A., Tinahones F.J. (2016). Biomarkers of Morbid Obesity and Prediabetes by Metabolomic Profiling of Human Discordant Phenotypes. Clin. Chim. Acta.

[93] Troisi J., Pierri L., Landolfi A., Marciano F., Bisogno A., Belmonte F., Palladino C., Guercio Nuzio S., Campiglia P., Vajro P. (2017). Urinary Metabolomics in Pediatric Obesity and NAFLD Identifies Metabolic Pathways/Metabolites Related to Dietary Habits and Gut-Liver Axis Perturbations. Nutrients.

[94] Fattuoni C., Mando C., Palmas F., Anelli G.M., Novielli C., Parejo Laudicina E., Savasi V.M., Barberini L., Dessi A., Pintus R. (2018). Preliminary metabolomics analysis of placenta in maternal obesity. Placenta.

[95] Bagheri M., Farzadfar F., Qi L., Yekaninejad M.S., Chamari M., Zeleznik O.A., Kalantar Z., Ebrahimi Z., Sheidaie A., Koletzko B. (2018). Obesity-Related Metabolomic Profiles and Discrimination of Metabolically Unhealthy Obesity. J. Proteome Res..

[96] Bervoets L., Massa G., Guedens W., Reekmans G., Noben J.P., Adriaensens P. (2018). Identification of metabolic phenotypes in childhood obesity by (1)H NMR metabolomics of blood plasma. Future Sci. OA.

[97] Libert D.M., Nowacki A.S., Natowicz M.R. (2018). Metabolomic analysis of obesity, metabolic syndrome, and type 2 diabetes: Amino acid and acylcarnitine levels change along a spectrum of metabolic wellness. PeerJ.

[98] Yu H.T., Fu X.Y., Xu B., Zuo L.L., Ma H.B., Wang S.R. (2018). Untargeted metabolomics approach (UPLC-Q-TOF-MS) explores the biomarkers of serum and urine in overweight/obese young men. Asia Pac. J. Clin. Nutr..

[99] Bagheri M., Djazayery A., Farzadfar F., Qi L., Yekaninejad M.S., Aslibekyan S., Chamari M., Hassani H., Koletzko B., Uhl O. (2019). Plasma metabolomic profiling of amino acids and polar lipids in Iranian obese adults. Lipids Health Dis..

[100] Kim M.J., Kim J.H., Kim M.S., Yang H.J., Lee M., Kwon D.Y. (2019). Metabolomics Associated with Genome-Wide Association Study Related to the Basal Metabolic Rate in Overweight/Obese Korean Women. J. Med. Food.

[101] Bellissimo M.P., Cai Q., Ziegler T.R., Liu K.H., Tran P.H., Vos M.B., Martin G.S., Jones D.P., Yu T., Alvarez J.A. (2019). Plasma High-Resolution Metabolomics Differentiates Adults with Normal Weight Obesity from Lean Individuals. Obesity.

[102] Lokhov P.G., Balashova E.E., Trifonova O.P., Maslov D.L., Ponomarenko E.A., Archakov A.I. (2020). Mass Spectrometry-Based Metabolomics Analysis of Obese Patients’ Blood Plasma. Int. J. Mol. Sci..

[103] Zhou B., Ichikawa R., Parnell L.D., Noel S.E., Zhang X., Bhupathiraju S.N., Smith C.E., Tucker K.L., Ordovas J.M., Lai C.Q. (2020). Metabolomic Links between Sugar-Sweetened Beverage Intake and Obesity. J. Obes..

[104] Hsu Y.H., Astley C.M., Cole J.B., Vedantam S., Mercader J.M., Metspalu A., Fischer K., Fortney K., Morgen E.K., Gonzalez C. (2020). Integrating untargeted metabolomics, genetically informed causal inference, and pathway enrichment to define the obesity metabolome. Int. J. Obes..

[105] Zhou L., Ni Z., Yu J., Cheng W., Cai Z., Yu C. (2020). Correlation Between Fecal Metabolomics and Gut Microbiota in Obesity and Polycystic Ovary Syndrome. Front. Endocrinol..

[106] Barlow S.E., Dietz W.H. (1998). Obesity evaluation and treatment: Expert Committee recommendations. The Maternal and Child Health Bureau, Health Resources and Services Administration and the Department of Health and Human Services. Pediatrics.

[107] Gall W.E., Beebe K., Lawton K.A., Adam K.P., Mitchell M.W., Nakhle P.J., Ryals J.A., Milburn M.V., Nannipieri M., Camastra S. (2010). alpha-hydroxybutyrate is an early biomarker of insulin resistance and glucose intolerance in a nondiabetic population. PLoS ONE.

[108] Bulut O., Temba G.S., Koeken V., Moorlag S., de Bree L.C.J., Mourits V.P., Kullaya V.I., Jaeger M., Qi C., Riksen N.P. (2024). Common and distinct metabolomic markers related to immune aging in Western European and East African populations. Mech. Ageing Dev..

[109] Feng R., Tian Z., Mao R., Ma R., Luo W., Zhao M., Li X., Liu Y., Huang K., Xiang L. (2023). Gut Microbiome-Generated Phenylacetylglutamine from Dietary Protein is Associated with Crohn’s Disease and Exacerbates Colitis in Mouse Model Possibly via Platelet Activation. J. Crohns Colitis.

[110] Sibal L., Agarwal S.C., Home P.D., Boger R.H. (2010). The Role of Asymmetric Dimethylarginine (ADMA) in Endothelial Dysfunction and Cardiovascular Disease. Curr. Cardiol. Rev..

[111] Nunez-Sanchez M.A., Martinez-Sanchez M.A., Sierra-Cruz M., Lambertos A., Rico-Chazarra S., Oliva-Bolarin A., Balaguer-Roman A., Yuste J.E., Martinez C.M., Mika A. (2024). Increased hepatic putrescine levels as a new potential factor related to the progression of metabolic dysfunction-associated steatotic liver disease. J. Pathol..

[112] Tan S.T., Ramesh T., Toh X.R., Nguyen L.N. (2020). Emerging roles of lysophospholipids in health and disease. Prog. Lipid Res..

[113] Kalhan S.C., Guo L., Edmison J., Dasarathy S., McCullough A.J., Hanson R.W., Milburn M. (2011). Plasma metabolomic profile in nonalcoholic fatty liver disease. Metabolism.

[114] Manna S.K., Patterson A.D., Yang Q., Krausz K.W., Li H., Idle J.R., Fornace A.J., Gonzalez F.J. (2010). Identification of noninvasive biomarkers for alcohol-induced liver disease using urinary metabolomics and the Ppara-null mouse. J. Proteome Res..

[115] Manna S.K., Patterson A.D., Yang Q., Krausz K.W., Idle J.R., Fornace A.J., Gonzalez F.J. (2011). UPLC-MS-based urine metabolomics reveals indole-3-lactic acid and phenyllactic acid as conserved biomarkers for alcohol-induced liver disease in the Ppara-null mouse model. J. Proteome Res..

[116] Shi X., Wei X., Yin X., Wang Y., Zhang M., Zhao C., Zhao H., McClain C.J., Feng W., Zhang X. (2015). Hepatic and fecal metabolomic analysis of the effects of Lactobacillus rhamnosus GG on alcoholic fatty liver disease in mice. J. Proteome Res..

[117] Lai Y.S., Chen W.C., Kuo T.C., Ho C.T., Kuo C.H., Tseng Y.J., Lu K.H., Lin S.H., Panyod S., Sheen L.Y. (2015). Mass-Spectrometry-Based Serum Metabolomics of a C57BL/6J Mouse Model of High-Fat-Diet-Induced Non-alcoholic Fatty Liver Disease Development. J. Agric. Food Chem..

[118] Jin R., Banton S., Tran V.T., Konomi J.V., Li S., Jones D.P., Vos M.B. (2016). Amino Acid Metabolism is Altered in Adolescents with Nonalcoholic Fatty Liver Disease-An Untargeted, High Resolution Metabolomics Study. J. Pediatr..

[119] Wang Y., Niu M., Jia G.L., Li R.S., Zhang Y.M., Zhang C.E., Meng Y.K., Cui H.R., Ma Z.J., Li D.H. (2016). Untargeted Metabolomics Reveals Intervention Effects of Total Turmeric Extract in a Rat Model of Nonalcoholic Fatty Liver Disease. Evid. Based Complement. Alternat Med..

[120] Koch M., Freitag-Wolf S., Schlesinger S., Borggrefe J., Hov J.R., Jensen M.K., Pick J., Markus M.R.P., Hopfner T., Jacobs G. (2017). Serum metabolomic profiling highlights pathways associated with liver fat content in a general population sample. Eur. J. Clin. Nutr..

[121] Han J., Dzierlenga A.L., Lu Z., Billheimer D.D., Torabzadeh E., Lake A.D., Li H., Novak P., Shipkova P., Aranibar N. (2017). Metabolomic profiling distinction of human nonalcoholic fatty liver disease progression from a common rat model. Obesity.

[122] Dong S., Zhan Z.Y., Cao H.Y., Wu C., Bian Y.Q., Li J.Y., Cheng G.H., Liu P., Sun M.Y. (2017). Urinary metabolomics analysis identifies key biomarkers of different stages of nonalcoholic fatty liver disease. World J. Gastroenterol..

[123] Tu L.N., Showalter M.R., Cajka T., Fan S., Pillai V.V., Fiehn O., Selvaraj V. (2017). Metabolomic characteristics of cholesterol-induced non-obese nonalcoholic fatty liver disease in mice. Sci. Rep..

[124] Prisingkorn W., Prathomya P., Jakovlic I., Liu H., Zhao Y.H., Wang W.M. (2017). Transcriptomics, metabolomics and histology indicate that high-carbohydrate diet negatively affects the liver health of blunt snout bream (*Megalobrama amblycephala*). BMC Genom..

[125] Romero-Ibarguengoitia M.E., Vadillo-Ortega F., Caballero A.E., Ibarra-Gonzalez I., Herrera-Rosas A., Serratos-Canales M.F., Leon-Hernandez M., Gonzalez-Chavez A., Mummidi S., Duggirala R. (2018). Family history and obesity in youth, their effect on acylcarnitine/aminoacids metabolomics and non-alcoholic fatty liver disease (NAFLD). Structural equation modeling approach. PLoS ONE.

[126] Mayo R., Crespo J., Martinez-Arranz I., Banales J.M., Arias M., Minchole I., Aller de la Fuente R., Jimenez-Aguero R., Alonso C., de Luis D.A. (2018). Metabolomic-based noninvasive serum test to diagnose nonalcoholic steatohepatitis: Results from discovery and validation cohorts. Hepatol. Commun..

[127] Yang Z., Kusumanchi P., Ross R.A., Heathers L., Chandler K., Oshodi A., Thoudam T., Li F., Wang L., Liangpunsakul S. (2019). Serum Metabolomic Profiling Identifies Key Metabolic Signatures Associated With Pathogenesis of Alcoholic Liver Disease in Humans. Hepatol. Commun..

[128] Gawlik A., Shmoish M., Hartmann M.F., Wudy S.A., Olczak Z., Gruszczynska K., Hochberg Z. (2019). Steroid metabolomic signature of liver disease in nonsyndromic childhood obesity. Endocr. Connect..

[129] Cui H., Li Y., Cao M., Liao J., Liu X., Miao J., Fu H., Song R., Wen W., Zhang Z. (2020). Untargeted Metabolomic Analysis of the Effects and Mechanism of Nuciferine Treatment on Rats With Nonalcoholic Fatty Liver Disease. Front. Pharmacol..

[130] Chang Y., Gao X.Q., Shen N., He J., Fan X., Chen K., Lin X.H., Li H.M., Tian F.S., Li H. (2020). A targeted metabolomic profiling of plasma acylcarnitines in nonalcoholic fatty liver disease. Eur. Rev. Med. Pharmacol. Sci..

[131] Lu Y., Shao M., Xiang H., Zheng P., Wu T., Ji G. (2020). Integrative transcriptomics and metabolomics explore the mechanism of kaempferol on improving nonalcoholic steatohepatitis. Food Funct..

[132] Zheng T.X., Pu S.L., Tan P., Du Y.C., Qian B.L., Chen H., Fu W.G., Huang M.Z. (2020). Liver Metabolomics Reveals the Effect of Lactobacillus reuteri on Alcoholic Liver Disease. Front. Physiol..

[133] Zhao M., Xing Y., Liu L., Fan X., Liu L., Geng T., Gong D. (2020). GC-TOF-MS-Based Metabolomics Analyses of Liver and Intestinal Contents in the Overfed vs. Normally-Fed Geese. Animals.

[134] Xue L.J., Han J.Q., Zhou Y.C., Peng H.Y., Yin T.F., Li K.M., Yao S.K. (2020). Untargeted metabolomics characteristics of nonobese nonalcoholic fatty liver disease induced by high-temperature-processed feed in Sprague-Dawley rats. World J. Gastroenterol..

[135] Shi C., Wang L., Zhou K., Shao M., Lu Y., Wu T. (2020). Targeted Metabolomics Identifies Differential Serum and Liver Amino Acids Biomarkers in Rats with Alcoholic Liver Disease. J. Nutr. Sci. Vitaminol..

[136] Masarone M., Troisi J., Aglitti A., Torre P., Colucci A., Dallio M., Federico A., Balsano C., Persico M. (2021). Untargeted metabolomics as a diagnostic tool in NAFLD: Discrimination of steatosis, steatohepatitis and cirrhosis. Metabolomics.

[137] Yang Y., Huang Z., Yang Z., Qi Y., Shi H., Zhou Y., Wang F., Yang M. (2021). Serum metabolomic profiling reveals an increase in homocitrulline in Chinese patients with nonalcoholic fatty liver disease: A retrospective study. PeerJ.

[138] Mazzini F.N., Cook F., Gounarides J., Marciano S., Haddad L., Tamaroff A.J., Casciato P., Narvaez A., Mascardi M.F., Anders M. (2021). Plasma and stool metabolomics to identify microbiota derived-biomarkers of metabolic dysfunction-associated fatty liver disease: Effect of PNPLA3 genotype. Metabolomics.

[139] Kordy K., Li F., Lee D.J., Kinchen J.M., Jew M.H., La Rocque M.E., Zabih S., Saavedra M., Woodward C., Cunningham N.J. (2021). Metabolomic Predictors of Non-alcoholic Steatohepatitis and Advanced Fibrosis in Children. Front. Microbiol..

[140] Feng Y., Li H., Chen C., Lin H., Xu G., Li H., Wang C., Chen J., Sun J. (2021). Study on the Hepatoprotection of Schisandra chinensis Caulis Polysaccharides in Nonalcoholic Fatty Liver Disease in Rats Based on Metabolomics. Front. Pharmacol..

[141] Mowry C.J., Alonso C., Iruarrizaga-Lejarreta M., Ortiz P., Levitsky J., Rinella M. (2021). Utility of Metabolomic Biomarkers to Identify Nonalcoholic Fatty Liver Disease in Liver Transplant Recipients. Transplant. Direct.

[142] He D., Su Y., Meng D., Wang X., Wang J., Ye H. (2021). A pilot study optimizing metabolomic and lipidomic acquisition in serum for biomarker discovery in nonalcoholic fatty liver disease. J. Mass. Spectrom. Adv. Clin. Lab..

[143] Zhu M.N., Zhao C.Z., Wang C.Z., Rao J.B., Qiu Y.W., Gao Y.P., Wang X.Y., Zhang Y.M., Wu G., Chen J. (2022). Dataset for liver metabolomic profile of highland barley Monascus purpureus went extract-treated golden hamsters with nonalcoholic fatty liver disease. Data Brief..

[144] Yan X., Li L., Liu P., Xu J., Wang Z., Ding L., Yang L. (2022). Targeted metabolomics profiles serum fatty acids by HFD induced non-alcoholic fatty liver in mice based on GC-MS. J. Pharm. Biomed. Anal..

[145] Charkoftaki G., Tan W.Y., Berrios-Carcamo P., Orlicky D.J., Golla J.P., Garcia-Milian R., Aalizadeh R., Thomaidis N.S., Thompson D.C., Vasiliou V. (2022). Liver metabolomics identifies bile acid profile changes at early stages of alcoholic liver disease in mice. Chem. Biol. Interact..

[146] Cao Y.J., Li H.Z., Zhao J., Sun Y.M., Jin X.W., Lv S.Q., Luo J.Y., Fang X.X., Wen W.B., Liao J.B. (2022). Mechanical Study of Jian-Gan-Xiao-Zhi Decoction on Nonalcoholic Fatty Liver Disease Based on Integrated Network Pharmacology and Untargeted Metabolomics. Evid. Based Complement. Alternat Med..

[147] Zhang X., Liu T., Hou X., Hu C., Zhang L., Wang S., Zhang Q., Shi K. (2022). Multi-Channel Metabolomics Analysis Identifies Novel Metabolite Biomarkers for the Early Detection of Fatty Liver Disease in Dairy Cows. Cells.

[148] Shao L., Liu J., Song Y., Yang W., Gong L., Lyu Z., Zhu Q., Fu J., Li J., Shi J. (2023). Serum metabolomics-based heterogeneities and screening strategy for metabolic dysfunction-associated fatty liver disease (MAFLD). Clin. Chim. Acta.

[149] Demirel M., Koktasoglu F., Ozkan E., Dulun Agac H., Gul A.Z., Sharifov R., Sarikaya U., Basaranoglu M., Selek S. (2023). Mass spectrometry-based untargeted metabolomics study of non-obese individuals with non-alcoholic fatty liver disease. Scand. J. Gastroenterol..

[150] Gagnon E., Manikpurage H.D., Mitchell P.L., Girard A., Gobeil E., Bourgault J., Begin F., Marette A., Theriault S., Arsenault B.J. (2023). Large-scale metabolomic profiling and incident non-alcoholic fatty liver disease. iScience.

[151] Garibay-Nieto N., Pedraza-Escudero K., Omana-Guzman I., Garces-Hernandez M.J., Villanueva-Ortega E., Flores-Torres M., Perez-Hernandez J.L., Leon-Hernandez M., Laresgoiti-Servitje E., Palacios-Gonzalez B. (2023). Metabolomic Phenotype of Hepatic Steatosis and Fibrosis in Mexican Children Living with Obesity. Medicina.

[152] Calzadilla N., Zilberstein N., Hanscom M., Al Rashdan H.T., Chacra W., Gill R.K., Alrefai W.A. (2024). Serum metabolomic analysis in cirrhotic alcohol-associated liver disease patients identified differentially altered microbial metabolites and novel potential biomarkers for disease severity. Dig. Liver Dis..

[153] Xu J., Jin Y., Song C., Chen G., Li Q., Yuan H., Wei S., Yang M., Li S., Jin S. (2023). Comparative analysis of the synergetic effects of Diwuyanggan prescription on high fat diet-induced non-alcoholic fatty liver disease using untargeted metabolomics. Heliyon.

[154] Thing M., Werge M.P., Kimer N., Hetland L.E., Rashu E.B., Nabilou P., Junker A.E., Galsgaard E.D., Bendtsen F., Laupsa-Borge J. (2024). Targeted metabolomics reveals plasma short-chain fatty acids are associated with metabolic dysfunction-associated steatotic liver disease. BMC Gastroenterol..

[155] Zhang F., Wu R., Liu Y., Dai S., Gong X., Li Y. (2024). Integration of pharmacodynamics and metabolomics to reveal rhubarb anthraquinone protection against nonalcoholic fatty liver disease rat model. J. Pharm. Pharmacol..

[156] Zhu Z., Zhang Y., Li J., Han Y., Wang L., Zhang Y., Geng H., Zheng Y., Wang X., Sun C. (2024). Mass spectrometry imaging-based metabolomics highlights spatial metabolic alterations in three types of liver injuries. J. Pharm. Biomed. Anal..

[157] Huneault H.E., Gent A.E., Cohen C.C., He Z., Jarrell Z.R., Kamaleswaran R., Vos M.B. (2024). Validation of a screening panel for pediatric metabolic dysfunction-associated steatotic liver disease using metabolomics. Hepatol. Commun..

[158] Luo J., Luo M., Kaminga A.C., Wei J., Dai W., Peng Y., Zhao K., Duan Y., Xiao X., Ouyang S. (2024). Integrative metabolomics highlights gut microbiota metabolites as novel NAFLD-related candidate biomarkers in children. Microbiol. Spectr..

[159] Tang N., Ji L., Shi X., Xiong Y., Xiong X., Zhao H., Song H., Wang J., Zhang L., You S. (2024). Effects of Ganjianglingzhu Decoction on Lean Non-Alcoholic Fatty Liver Disease in Mice Based on Untargeted Metabolomics. Pharmaceuticals.

[160] Daniels N.J., Hershberger C.E., Kerosky M., Wehrle C.J., Raj R., Aykun N., Allende D.S., Aucejo F.N., Rotroff D.M. (2024). Biomarker Discovery in Liver Disease Using Untargeted Metabolomics in Plasma and Saliva. Int. J. Mol. Sci..

[161] Zhang S., Peng X., Yang S., Li X., Huang M., Wei S., Liu J., He G., Zheng H., Yang L. (2022). The regulation, function, and role of lipophagy, a form of selective autophagy, in metabolic disorders. Cell Death Dis..

[162] Filali-Mouncef Y., Hunter C., Roccio F., Zagkou S., Dupont N., Primard C., Proikas-Cezanne T., Reggiori F. (2022). The menage a trois of autophagy, lipid droplets and liver disease. Autophagy.

[163] Kaini R.R., Sillerud L.O., Zhaorigetu S., Hu C.A. (2012). Autophagy regulates lipolysis and cell survival through lipid droplet degradation in androgen-sensitive prostate cancer cells. Prostate.

[164] Korbecki J., Bosiacki M., Kupnicka P., Barczak K., Zietek P., Chlubek D., Baranowska-Bosiacka I. (2024). Biochemistry and Diseases Related to the Interconversion of Phosphatidylcholine, Phosphatidylethanolamine, and Phosphatidylserine. Int. J. Mol. Sci..

[165] Sun C., Holstein D.J.F., Garcia-Cubero N., Moulla Y., Stroh C., Dietrich A., Schon M.R., Gartner D., Lohmann T., Dressler M. (2023). The Role of Phosphatidylethanolamine N-Methyltransferase (PEMT) and Its Waist-Hip-Ratio-Associated Locus rs4646404 in Obesity-Related Metabolic Traits and Liver Disease. Int. J. Mol. Sci..

[166] Song J., da Costa K.A., Fischer L.M., Kohlmeier M., Kwock L., Wang S., Zeisel S.H. (2005). Polymorphism of the PEMT gene and susceptibility to nonalcoholic fatty liver disease (NAFLD). FASEB J..

[167] Wan S., van der Veen J.N., Bakala N’Goma J.C., Nelson R.C., Vance D.E., Jacobs R.L. (2019). Hepatic PEMT activity mediates liver health, weight gain, and insulin resistance. FASEB J..

[168] Alonso C., Fernandez-Ramos D., Varela-Rey M., Martinez-Arranz I., Navasa N., Van Liempd S.M., Lavin Trueba J.L., Mayo R., Ilisso C.P., de Juan V.G. (2017). Metabolomic Identification of Subtypes of Nonalcoholic Steatohepatitis. Gastroenterology.

[169] Zurier R.B., Burstein S.H. (2016). Cannabinoids, inflammation, and fibrosis. FASEB J..

[170] Kasatkina L.A., Rittchen S., Sturm E.M. (2021). Neuroprotective and Immunomodulatory Action of the Endocannabinoid System under Neuroinflammation. Int. J. Mol. Sci..

[171] Forlani L., Cristoni G., Boga C., Todesco T.E., Del Vecchio E., Selva S., Monari M. (2002). Reinvestigation of the tautomerism of some substituted 2-hydroxypyridines. ARKIVOC.

[172] Neubert P., Holck J.P. (1989). Automated pre-column high-performance liquid chromatographic method for the investigation of adibendan metabolism. J. Chromatogr..

[173] Denno M.E., Privman E., Borman R.P., Wolin D.C., Venton B.J. (2016). Quantification of Histamine and Carcinine in Drosophila melanogaster Tissues. ACS Chem. Neurosci..

[174] Boeglin W.E., Kim R.B., Brash A.R. (1998). A 12R-lipoxygenase in human skin: Mechanistic evidence, molecular cloning, and expression. Proc. Natl. Acad. Sci. USA.

[175] Mashima R., Okuyama T. (2015). The role of lipoxygenases in pathophysiology; new insights and future perspectives. Redox Biol..

[176] Aguilera J.A., Linares A., Arce V., Garcia-Peregrin E. (1983). Incorporation of mevalonate into squalene, lanosterol and cholesterol by different neonatal chick tissues. Int. J. Biochem..

[177] Chua N.K., Coates H.W., Brown A.J. (2020). Squalene monooxygenase: A journey to the heart of cholesterol synthesis. Prog. Lipid Res..

[178] Li Z., Agellon L.B., Allen T.M., Umeda M., Jewell L., Mason A., Vance D.E. (2006). The ratio of phosphatidylcholine to phosphatidylethanolamine influences membrane integrity and steatohepatitis. Cell Metab..

[179] van der Veen J.N., Kennelly J.P., Wan S., Vance J.E., Vance D.E., Jacobs R.L. (2017). The critical role of phosphatidylcholine and phosphatidylethanolamine metabolism in health and disease. Biochim. Biophys. Acta Biomembr..

[180] Vance D.E. (2014). Phospholipid methylation in mammals: From biochemistry to physiological function. Biochim. Biophys. Acta.

[181] Lopez M.J., Mohiuddin S.S. (2024). Biochemistry, Essential Amino Acids. StatPearls.

[182] Shen J., Xie E., Shen S., Song Z., Li X., Wang F., Min J. (2024). Essentiality of SLC7A11-Mediated Nonessential Amino Acids in MASLD. Sci. Bull..

[183] Gobeil E., Maltais-Payette I., Taba N., Briere F., Ghodsian N., Abner E., Bourgault J., Gagnon E., Manikpurage H.D., Couture C. (2022). Mendelian Randomization Analysis Identifies Blood Tyrosine Levels as a Biomarker of Non-Alcoholic Fatty Liver Disease. Metabolites.

[184] Masoodi M., Gastaldelli A., Hyotylainen T., Arretxe E., Alonso C., Gaggini M., Brosnan J., Anstee Q.M., Millet O., Ortiz P. (2021). Metabolomics and lipidomics in NAFLD: Biomarkers and non-invasive diagnostic tests. Nat. Rev. Gastroenterol. Hepatol..

[185] Moore M.P., Shryack G., Alessi I., Wieschhaus N., Meers G.M., Johnson S.A., Wheeler A.A., Ibdah J.A., Parks E.J., Rector R.S. (2024). Relationship between serum beta-hydroxybutyrate and hepatic fatty acid oxidation in individuals with obesity and NAFLD. Am. J. Physiol. Endocrinol. Metab..

[186] Post A., Garcia E., van den Berg E.H., Flores-Guerrero J.L., Gruppen E.G., Groothof D., Westenbrink B.D., Connelly M.A., Bakker S.J.L., Dullaart R.P.F. (2021). Nonalcoholic fatty liver disease, circulating ketone bodies and all-cause mortality in a general population-based cohort. Eur. J. Clin. Investig..

[187] Nunez-Sanchez M.A., Martinez-Sanchez M.A., Martinez-Montoro J.I., Balaguer-Roman A., Murcia-Garcia E., Fernandez-Ruiz V.E., Ferrer-Gomez M., Martinez-Caceres C.M., Sledzinski T., Frutos M.D. (2024). Lipidomic Analysis Reveals Alterations in Hepatic FA Profile Associated With MASLD Stage in Patients With Obesity. J. Clin. Endocrinol. Metab..

[188] Morales-Marroquin E., Hanson B., Greathouse L., de la Cruz-Munoz N., Messiah S.E. (2020). Comparison of methodological approaches to human gut microbiota changes in response to metabolic and bariatric surgery: A systematic review. Obes. Rev..

[189] Steffen K.J., Sorgen A.A., Fodor A.A., Carroll I.M., Crosby R.D., Mitchell J.E., Bond D.S., Heinberg L.J. (2024). Early changes in the gut microbiota are associated with weight outcomes over 2 years following metabolic and bariatric surgery. Obesity.

[190] Beyoglu D., Idle J.R. (2022). The gut microbiota—A vehicle for the prevention and treatment of hepatocellular carcinoma. Biochem. Pharmacol..

[191] Dave A., Beyoglu D., Park E.J., Idle J.R., Pezzuto J.M. (2023). Influence of grape consumption on the human microbiome. Sci. Rep..

[192] Dave A., Park E.J., Kumar A., Parande F., Beyoglu D., Idle J.R., Pezzuto J.M. (2022). Consumption of Grapes Modulates Gene Expression, Reduces Non-Alcoholic Fatty Liver Disease, and Extends Longevity in Female C57BL/6J Mice Provided with a High-Fat Western-Pattern Diet. Foods.

[193] Queathem E.D., Stagg D.B., Nelson A.B., Chaves A.B., Crown S.B., Fulghum K., d’Avignon D.A., Ryder J.R., Bolan P.J., Hayir A. (2024). Ketogenesis protects against MASLD-MASH progression through fat oxidation-independent mechanisms. bioRxiv.

[194] Fernandez-Tussy P., Cardelo M.P., Zhang H., Sun J., Price N.L., Boutagy N.E., Goedeke L., Cadena-Sandoval M., Xirouchaki C.E., Brown W. (2024). miR-33 deletion in hepatocytes attenuates MASLD-MASH-HCC progression. JCI Insight.

[195] Biro F.M., Wien M. (2010). Childhood obesity and adult morbidities. Am. J. Clin. Nutr..

[196] Hines R.N. (2007). Ontogeny of human hepatic cytochromes P450. J. Biochem. Mol. Toxicol..

[197] Subash S., Singh D.K., Ahire D., Khojasteh S.C., Murray B.P., Zientek M.A., Jones R.S., Kulkarni P., Zubair F., Smith B.J. (2024). Ontogeny of Human Liver Aldehyde Oxidase: Developmental Changes and Implications for Drug Metabolism. Mol. Pharm..

[198] Badee J., Qiu N., Collier A.C., Takahashi R.H., Forrest W.F., Parrott N., Schmidt S., Fowler S. (2019). Characterization of the Ontogeny of Hepatic UDP-Glucuronosyltransferase Enzymes Based on Glucuronidation Activity Measured in Human Liver Microsomes. J. Clin. Pharmacol..

